# LUCas: Light-Uncaged Cas13a using photocleavable interfering guide RNAs

**DOI:** 10.1093/nar/gkag848

**Published:** 2026-08-31

**Authors:** Carlos F Ng, Deepak Krishnamurthy, Andres Dextre, Aymeric Chorlay, Melanie Ott, Daniel A Fletcher

**Affiliations:** Department of Bioengineering, University of California, Berkeley, CA 94720, United States; UC Berkeley-UC San Francisco Graduate Program in Bioengineering, University of California, Berkeley, CA 94720, United States; Department of Bioengineering, University of California, Berkeley, CA 94720, United States; Department of Bioengineering, University of California, Berkeley, CA 94720, United States; UC Berkeley-UC San Francisco Graduate Program in Bioengineering, University of California, Berkeley, CA 94720, United States; Department of Bioengineering, University of California, Berkeley, CA 94720, United States; Chan Zuckerberg Biohub, San Francisco, CA 94158, United States; Gladstone Infectious Disease Institute, Gladstone Institutes, San Francisco, CA 94158, United States; Department of Medicine, University of California, San Francisco, CA 94158, United States; Department of Bioengineering, University of California, Berkeley, CA 94720, United States; UC Berkeley-UC San Francisco Graduate Program in Bioengineering, University of California, Berkeley, CA 94720, United States; Chan Zuckerberg Biohub, San Francisco, CA 94158, United States; Biophysics Program, University of California, Berkeley, CA 94720, United States; Division of Biological Systems and Engineering, Lawrence Berkeley National Laboratory, Berkeley, CA 94720, United States

## Abstract

CRISPR diagnostics enable sensitive detection of infectious diseases, with the RNA endonuclease Cas13a providing specific, amplification-free RNA detection through collateral *trans*-cleavage of fluorescent reporters. However, background cleavage from unbound enzyme, contaminating nucleases, and unsynchronized initiation of reactions limits assay sensitivity and interpretability. A strategy to precisely control the onset of Cas13a catalytic activity, essentially a molecular “starting gun,” would address these challenges. Here, we introduce Light-Uncaged Cas13a (LUCas), a light-controllable system that directly blocks Cas13a *trans*-cleavage activity using a photocleavable interfering guide RNA, even in the presence of target RNA. Brief UV illumination releases this suppression, restoring full activity. Quantitative kinetic analysis reveals an ~100-fold suppression of *trans*-cleavage activity prior to photo-uncaging, including suppression of target-independent background activity. Using measured kinetic parameters, we predict and experimentally validate the limit of detection of the LUCas system for direct detection. We further demonstrate a multiplexed detection strategy termed “temporal barcoding,” enabling quantitative detection of viral co-infections in a single bulk reaction. Finally, LUCas is shown to be compatible with one-pot isothermal amplification for enhanced sensitivity and direct detection of target RNA spiked into blood plasma. Together, these results establish LUCas as a general framework for mechanistically informed, light-based control of Cas13a activity.

## Graphical Abstract

**Graphical Abstract:**
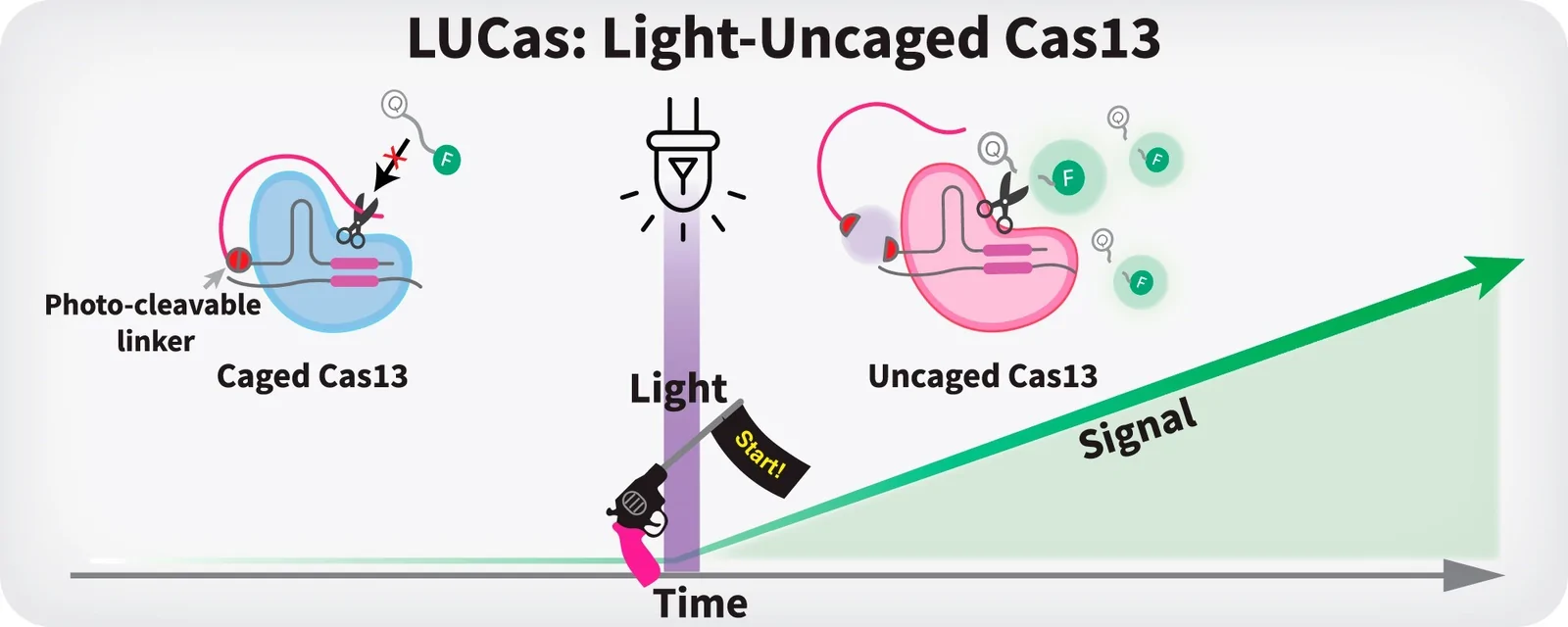

## Introduction

Clustered Regularly Interspaced Short Palindromic Repeats (CRISPR)-based diagnostics have demonstrated great potential for sensitive and specific detection of DNA and RNA targets over the last decade. In particular, the CRISPR enzymes Cas12a and Cas13a have been used to develop a range of diagnostic platforms such as SHERLOCK, DETECTR, SATORI, and CARMEN to detect nucleic acids from various pathogens and other targets [1–4]. When Cas13a, a Class 2 type VI enzyme, complexes with a guide RNA (gRNA), it forms a ribonucleoprotein (RNP). The gRNA is a single-stranded RNA (ssRNA) containing a stem-loop structure important for enzyme recognition and a seed region complementary to a target of interest. Subsequent binding of target RNA to the seed region of the RNP results in a conformational change in Cas13a that unleashes non-specific cleavage of ssRNA, which is known as *trans*-cleavage activity. This *trans*-cleavage activity is mediated by two conserved HEPN (higher eukaryotes and prokaryotes nucleotide-binding) domains located on the solvent-accessible surface of Cas13a [1, 2, 5]. For diagnostic applications, the *trans*-cleavage activity is leveraged by adding a reporter molecule that consists of a fluorophore–quencher pair connected through a short ssRNA sequence to the reaction. Upon target recognition by the gRNA, the RNP cleaves surrounding reporter molecules, resulting in an increase of fluorescence signal over time at a rate proportional to the target concentration [6].

To correctly interpret the signals generated in Cas13a in diagnostic assays, it is necessary to account for the various sources of noise that affect its signal-to-background ratio (SBR). For example, there is a background rate of cleavage of the reporter molecules even in the absence of a specific target RNA that can limit assay sensitivity. Cas13a reactions start as soon as a target RNA is bound, meaning that signal is being generated as soon as reagents are mixed together rather than when all target RNA has bound a Cas13a RNP and data collection is ready to begin. In addition, the uncontrolled nature of the amplification makes it challenging to distinguish the target-generated signal from that due to background nuclease activity. Lastly, when combined with pre-amplification techniques, the *trans*-cleavage activity of Cas13a can degrade intermediate products and hamper the detection sensitivity, thus requiring precise fluidic operations to mix the reaction at specific times [7–9].

Exploiting the full potential of Cas13a and other CRISPR/Cas enzymes for clinical applications requires strategies that can precisely control its activity in both space and time. In particular, a “starting-gun” for the *trans*-cleavage activity would be of practical value in designing and implementing diagnostic assays. To implement such a triggered initiation of the reaction, light-based control strategies have several key advantages: they are simple to implement, require no addition of reagents, and are highly tunable both in time and space.

Recently, there has been work demonstrating photo-control for Cas12a, motivated by the need to combine nucleic acid amplification methods with Cas12a-based detection in a single, one-pot assay without any fluid transfer steps [7, 10–13]. In that case, activating Cas12a reactions at a specific time prevents the *cis* and *trans*-cleavage activity from degrading the primers and products of the isothermal nucleic acid amplification steps, which would negatively impact the assay sensitivity [11]. The photo-control strategies developed for Cas12a involve the use of photocleavable “protective” or “silent” oligos that are complementary to the gRNA hairpin structure [14, 15], spacer region [16], or both regions [7, 11, 17]. This prevents RNP formation and/or target binding, stalling the Cas12a reaction until light exposure occurs. Other approaches for controlling enzyme activity focus on the gRNA, which can be a more universal strategy. For example, some studies have demonstrated light-based control by embedding photocaged groups, creating a “cloaking” effect that prevents target binding [10, 18–21] or caging specific nucleotides in the repeat recognition sequence [22]. Other studies have extended the 3′ end of the gRNA for Cas12a with a steric hindrance effector [23] or an inhibitory extension [12]. Recently, allosteric control of Cas13d catalytic activity using light was demonstrated [24], as was a photoactivatable split Cas13 system for RNA base editing [25]. However, currently, a simple, light-based method for Cas13a that directly modulates enzyme *trans*-cleavage and is independent of RNP formation and guide-target kinetics is still lacking.

Here, we present LUCas (Light-Uncaged Cas13a), a direct and modular photo-control strategy for Cas13a based on photocleavable interfering guide RNAs (pc-igRNAs). A pc-igRNA consists of a standard gRNA appended at its 5′ end with a single photocleavable linker followed by a short DNA-interfering segment that suppresses *trans*-cleavage even in the presence of target RNA. Importantly, this design decouples control of enzyme activity from RNP assembly and guide–target binding. Through quantitative kinetic analysis, we show that Cas13a with pc-igRNAs operates in a well-defined suppressed state prior to illumination and transitions to a fully active, uncaged state upon light exposure. We introduce a phenomenological “suppression factor” that captures the fold-change in *trans*-cleavage activity upon photo-uncaging. We demonstrate that this factor can be independently tuned by the length of the inhibitory DNA segment and the applied light dose. Notably, background cleavage is also suppressed before uncaging, enabling both predictive and experimental determination of the assay limit-of-detection, which we find to be set by intrinsic enzyme background activity.

To probe the mechanism underlying pc-igRNA-mediated control, we use fluorescence anisotropy to track complex formation between Cas13a and pc-igRNAs and determine how photo-uncaging rapidly restores full Cas13a *trans*-cleavage activity. We then leverage light as a temporal control knob to introduce “temporal barcoding,” a multiplexed Cas13a detection strategy in which distinct targets are encoded by their activation times rather than by spatial or spectral separation. Finally, we show that LUCas can be integrated into practical diagnostic workflows by combining it with RPA in a single reaction mix and demonstrating robust light-controlled activity in complex sample matrices such as plasma.

## Materials and methods

### Protein purification

Protein purification was performed as previously described [26]. The LbuCas13a expression vector contains the codon-optimized Cas13a genomic sequence, N-terminal His6-MBP-TEV cleavage site sequence, and a T7 promoter-binding sequence (Addgene Plasmid #83482). The protein is expressed in Rosetta 2 (DE3) pLysS *E. coli* cells in Terrific broth at $16\mathrm{^{\circ }C}$ overnight. Soluble His6-MBP-TEV-Cas13a was isolated over metal ion affinity chromatography and the His6-MBP tag was cleaved with TEV protease at $4\mathrm{^{\circ }C}$ overnight. Cleaved Cas13a was loaded onto a HiTrap SP column (GE Healthcare) and eluted over a linear KCl (0.25–1.0 M) gradient. Cas13a-containing fractions were further purified via size-exclusion chromatography on a S200 column (GE Healthcare) in gel filtration buffer (20 mM HEPES-K pH 7.0, 200 mM KCl, 10% glycerol, 1 mM TCEP) and subsequently flash frozen for storage at $-80\mathrm{^{\circ }C}$.

### Guide RNA and RPA primer design

All gRNAs, RPA primers, and pc-igRNAs were ordered from Synthego and Integrated DNA Technologies at a scale of 4 nmole (Supplementary Table S1). The photocleavable spacer is based on an ortho-nitrobenzyl linker, whose chemical structure is shown in (Supplementary Fig. S1) [27]. The spacer sequence was designed to recognize the SARS-CoV-2 N gene as previously described [26, 28]. The RPA primers used to amplify the SARS-CoV-2 N gene were previously published [7], but modified by adding a T7 promoter sequence to the 5′ end of the forward primer, which is the same strategy used for SHINE [29].

### Bulk Cas13a assay

Reactions were prepared by preassembling LbuCas13a-crRNA RNP complexes at 133 nM equimolar concentrations for 5 min at room temperature. After that, LbuCas13a is diluted to 25 nM in cleavage buffer (20 mM HEPES-Na pH 7.2, 50 mM KCl, 5 mM MgCl$_{2}$ and 5% glycerol) with the addition of 400 nM of a polyU reporter molecule, 1 unit/*μ*l (U/*μ*l) Murine RNase Inhibitor (NEB, Cat#M0314), diethylpyrocarbonate (DEPC)-treated water (Fisher Scientific, Cat#BP561-1), and varying amounts of target concentrations, yielding a 20 *μ*l final reaction volume. Once the reagents were mixed, the reactions were loaded into a 384-well (Corning, Cat#3544) and incubated in a plate reader (TECAN, Spark) for 60 min at $37\mathrm{^{\circ }C}$ with fluorescence intensity measurements obtained every 2.5 min ($\lambda _{ex}$: 485 nm; $\lambda _{em}$: 535 nm). A calibration curve (Supplementary Fig. S2) was used to convert the relative fluorescence unit (RFU) to cleaved reporter concentration (in nM) as previously done [30]. The reaction rate (in nM/sec) is obtained by fitting a linear regression or the Michaelis–Menten equation to the cleaved reporter concentration from the initial time to the reaction duration time. For reactions using photocleavable guides, the “pre-stim” data were measured during the first 15–30 min of the reaction, followed by the “post-stim” data measured for 60 min or more. For removing the cleaved DNA fragments, 0.125 U/*μ*l of DNase I solution (Thermo Fisher Scientific, Cat#90083) was added to the reaction.

### UV stimulation apparatus

For UV stimulation, a 365 nm high-power 96-well LED array (Analytical, Lumidox II) was used to photo-uncage individual 20-*μ*l CRISPR reactions (Supplementary Fig. S3A). To apply a high UV dose in a 384-well plate, a custom acrylic plate adapter was used to align each of the 96 LEDs to be on top of individual wells (Supplementary Fig. S3B and C). The 96-well LED array was connected to a controller unit that sets the power and stimulation time. A complete characterization of the illumination wavelengths and power level applied to an individual well can be found in Supplementary Fig. S4A and B, respectively. The non-stimulated reactions were simply covered during activation by the custom adapter, which is designed to avoid premature activation in neighboring wells (Supplementary Fig. S5B). All measurements were conducted in triplicate.

### Kinetic measurements

Bulk Cas13a cleavage kinetics were measured by adding RNA targets at defined concentrations to reaction mixtures containing Cas13a RNPs and fluorescent RNA reporters. Fluorescence intensity arising from reporter cleavage was monitored continuously for 30 min to quantify pre-uncaging activity. Reactions were then initiated by a 60 s exposure to 365 nm UV light using a Lumidox II plate illuminator, followed by continued fluorescence measurements for an additional 60 min (post-uncaging phase).

Fluorescence time courses were converted to cleaved reporter concentration using calibration curves and analyzed using a biphasic Michaelis–Menten model that independently accounts for pre- and post-uncaging reaction regimes. Reaction rates were estimated from the fits to pre- and post-uncaging phases by extracting the time-scale (*τ*) predicted by the model-predicted cleaved reporter concentration curves.

Uncertainty in the estimated reaction rates was computed by combining two independent contributions: (i) variability across experimental replicates and (ii) uncertainty in the fitted model parameters. These contributions were combined in quadrature to yield the total uncertainty reported for each reaction rate.

### Background activity measurements

To quantify target-independent background activity, reactions were performed at increasing RNP concentrations ranging from 25 to 250 nM. To reliably measure reporter cleavage at the low expected background catalytic rates, a higher reporter concentration (2000 nM) was used in these assays. Reaction rates were estimated using the same analysis pipeline as for the bulk kinetic measurements (see the “Materials and methods” section), by converting fluorescence signals to cleaved reporter concentrations using calibration curves and performing linear fits to extract reaction rates.

### One-Pot RPA-LUCas assay

Reactions were prepared by mixing LbuCas13a with 19T-pc-igRNA 1 at 133 nM equimolar concentrations for 5 min at room temperature. For the RPA mixture, one RPA pellet from the TwistAmp Basic Kit (TwistDx, Cat#TABAS03KIT) was rehydrated by adding 29.5 *μ*l of primer-free rehydration buffer. Then, the following reagents were added: 0.25 *μ*M of each forward and reverse RPA primers, 5.9 *μ*l of the RPA mixture, 400 nM of polyU reporter molecules, 2 U/*μ*l of SuperScript IV reverse transcriptase (Thermo Fisher Scientific), 2 U/*μ*l of Hi-T7 RNA polymerase (NEB, Cat#M0658S), 1 U/*μ*l of RNase Inhibitor, 1 mM of each ribonucleotide solution set (NEB, Cat#N0450S) and DEPC-treated water. Finally, 14 mM of magnesium acetate, 2 *μ*l of synthetic SARS-CoV-2 RNA virus (Twist Bioscience, Cat#102019) and 25 nM of RNP were added, resulting in a 20 *μ*l final reaction volume. The reaction mixture was loaded onto a 384-well plate, and its fluorescence was measured using the plate reader for 30 min (pre-uncaging) and for 60 min (post-uncaging), as previously described.

### Contrived plasma samples

Plasma was isolated by centrifugation of 1 ml of whole blood obtained from a single donor, collected with acid citrate dextrose (ACD) anticoagulant (Innovative Research) at 1500 RCF for 10 min at $4\mathrm{^{\circ }C}$. The supernatant was collected, aliquoted, and stored at $-80\mathrm{^{\circ }C}$ until the day of the experiment. Isolated plasma was diluted 25% (v/v) in nuclease-free water and treated with Proteinase K (ThermoFisher, Cat#EO0491) to a final concentration of 50 *μ*g/ml. The treated plasma was subsequently incubated at $55\mathrm{^{\circ }C}$ for 15 min, followed by heat inactivation at $95\mathrm{^{\circ }C}$ for 5 min. The treated plasma was divided into two aliquots, where one was spiked with a synthetic target at a final concentration of $2 \times 10^{5}$ cp/*μ*l, and the other was retained as a negative control. Both samples were added to the LUCas reaction.

### Fluorescence anisotropy assay

The photocleavable interfering guides (pc-igRNA) were labeled with Rhodamine Red C2 Maleimide (ThermoFisher, Cat#R6029) using the 5′ EndTag DNA/RNA Labeling Kit (Vector Laboratories, CA). After that, the fluorescently labeled pc-igRNA concentration was measured using a NanoDrop spectrophotometer (ThermoFisher) and aliquoted. For the actual measurements, a CRISPR–Cas13 reaction was prepared by mixing LbuCas13a with a 5′ fluorescently labeled pc-igRNA using a ratio of 4:1. After a 5 min incubation at room temperature, the mixture was diluted to a final concentration of 25 nM of LbuCas13a assembled with a photocleavable guide in cleavage buffer with 100 nM target RNA. Reactions were loaded into a 384-well plate (Corning, Cat#3820) and incubated in a temperature-controlled plate reader (TECAN, Spark) for 60 min at $37\mathrm{^{\circ }C}$ with fluorescence polarization measurements taken every 2.5 min ($\lambda _{ex}$: 570 nm; $\lambda _{em}$: 635 nm) that were used to calculate anisotropy (r):


(1) \begin{eqnarray*}
r=\frac{I_{\parallel }-GI_{\perp }}{I_{\parallel }+2GI_{\perp }} ,
\end{eqnarray*}

where $I_{\parallel }$ and $I_{\perp }$ are the background-subtracted parallel and perpendicular intensities, and *G* is a correction factor obtained from measuring polarization for a solution of 25 nM free-floating Rhodamine in cleavage buffer. All measurements were conducted in triplicate.

For the simultaneous fluorescence and anisotropy measurements, the reactions were prepared as described previously, and an alternating sequence of fluorescence polarization and intensity measurements was done on the plate reader. For the single stimulation, four consecutive fluorescence intensity measurements ($\lambda _{ex}$: 360 nm; $\lambda _{em}$: 520 nm) were used, followed by a series of alternating fluorescence polarization and intensity measurements. For the pulsed stimulation, a single fluorescence intensity measurement ($\lambda _{ex}$: 360 nm; $\lambda _{em}$: 520 nm) was added after each alternating fluorescence polarization and intensity measurement. The instantaneous slope for each condition was calculated by measuring the slope between two consecutive points of the fluorescence intensity over their time difference.

## Results

### Design and demonstration of the LUCas system

**Figure 1.**
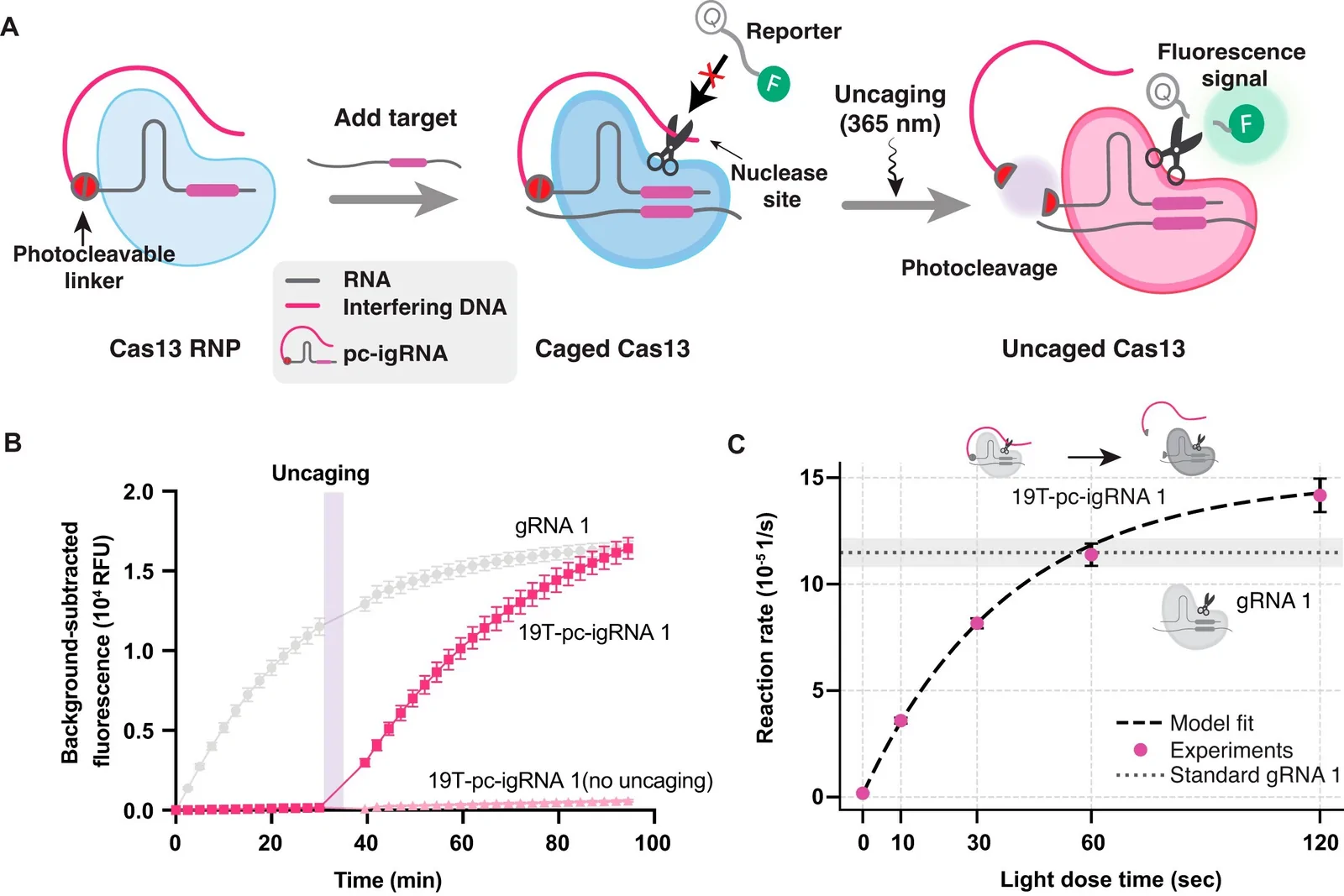
Demonstration of the LUCas system. (**A**) Schematic of the LUCas system for caging and light-triggered uncaging of Cas13a using a 19T photocleavable interfering guide RNA (19T-pc-igRNA). The pc-igRNA consists of a guide RNA (gRNA) linked at its 5′ end to a single-stranded DNA interfering segment via a photocleavable linker. (**B**) Photo-uncaging of Cas13a using a pc-igRNA containing a 19T interfering segment. Fluorescence increases due to reporter cleavage upon brief UV illumination (365 nm) in the presence of target RNA, whereas no UV exposure results in suppressed activity. Data points represent the mean across experimental replicates; error bars indicate standard error of the mean (SEM) (*n* = 3). (**C**) UV dose-dependent activation of Cas13a, demonstrating that the extent of photo-uncaging, and hence reaction rate, can be quantitatively and precisely tuned. For higher light doses, the rate saturates to the Cas13a reaction rate with standard guides (gray dotted line). Reaction rates were fitted with a model for photo-uncaging kinetics (black dashed curve); see Supplementary data. Data points and error bars represent the mean ± SEM for *n* = 3 wells.

In order to achieve light-based control of Cas13a activity, we designed a chimeric DNA–RNA photocleavable interfering guide (pc-igRNA). We previously introduced interfering guide RNAs (igRNA) that modulate the *trans*-cleavage activity of Cas13a through the addition of a 5′ DNA segment [31]. The photocleavable design consists of an interfering DNA segment linked to a canonical crRNA at the 5′ end via a photocleavable linker (Fig. 1A and Supplementary Fig. S1). Since a sequence of repeating thymine (T) nucleotide bases is most effective at suppressing *trans*-cleavage activity, we designed and tested interfering DNA segments consisting of a 19T sequence. Upon RNP complex formation with Cas13a from *Leptotrichia buccalis* (LbuCas13a) in the presence of target RNA, the DNA segment of the pc-igRNAs interferes with the *trans*-cleavage activity, leading to a “suppressed” state (Fig. 1A). When stimulated with an ultraviolet (UV) light dose at 365 nm, the resulting photocleavage releases the interfering DNA strand from the HEPN catalytic domain, leading to a photo-uncaged, active state (Fig. 1A).

We directly demonstrated both the caged and photo-uncaged states of the LUCas system by monitoring bulk fluorescence arising from cleavage of a fluorophore–quencher RNA reporter (5 uracils) [26] (Fig. 1B). In the presence of $10^{6}$ cp/*μ*l target RNA, a rapid increase in fluorescence was observed only in reactions receiving a 365 nm light dose, indicating light-triggered activation of Cas13a with pc-igRNA. In contrast, non-exposed reactions showed no measurable change in fluorescence, demonstrating strong suppression of *trans*-cleavage activity, even in the presence of target RNA (Fig. 1B). As expected, reactions programmed with standard guides exhibited high cleavage activity prior to UV exposure and were unaffected by UV illumination (Fig. 1B). Importantly, we established the generality of the LUCas strategy by reproducing robust suppression followed by photo-uncaging across multiple guide and target RNA combinations (Supplementary Fig. S6).

Importantly, using light provides a tunable control knob for Cas13a activity in the LUCas system, as both illumination intensity and duration can be precisely modulated to control *trans*-cleavage rate. We find that increasing the 365 nm light dose photoactivates a larger fraction of the RNP population, producing a monotonic, but saturating increase in reaction rate (Fig. 1C). This response saturates at ~60 s of UV exposure at an intensity of 16.1 mW/cm$^{2}$ (corresponding to an estimated total energy of 96 mJ per well), beyond which no further increase in activity is observed and the reaction rate is comparable to that of a standard guide (Fig. 1C).

To quantitatively describe this dose response, we modeled photo-uncaging as an irreversible first-order conversion of caged RNPs into uncaged RNPs and find that the uncaged fraction increases as a saturating exponential function of cumulative light dose (Supplementary data). Notably, in this model, the measured reaction rate is the weighted sum of the residual activity from the remaining caged RNPs and the restored activity from the uncaged RNPs. This simple model captures both the initial dose-dependent increase in activity and the high-dose saturation regime, showing excellent agreement with the experimental reaction rate data as a function of light dose in Fig. 1C. Together, these results demonstrate that light dose can be used to precisely and quantitatively tune the fraction of uncaged, target-bound RNPs and therefore the effective Cas13a *trans*-cleavage activity.

An additional consideration is that the DNA fragment released upon light-induced cleavage may retain a slight inhibitory effect on *trans*-cleavage activity (Supplementary Fig. S7A and B). Given the structural similarity between thymine and uracil, we hypothesize that the released DNA fragment competes with the reporter molecule for binding to the Cas13a active site. We find that this residual effect can be eliminated by adding DNase I, consistent with degradation of the released DNA fragment, while suppression by intact pc-igRNAs is preserved (Supplementary Fig. S7C).

### Characterization of enzyme *trans*-kinetics

**Figure 2.**
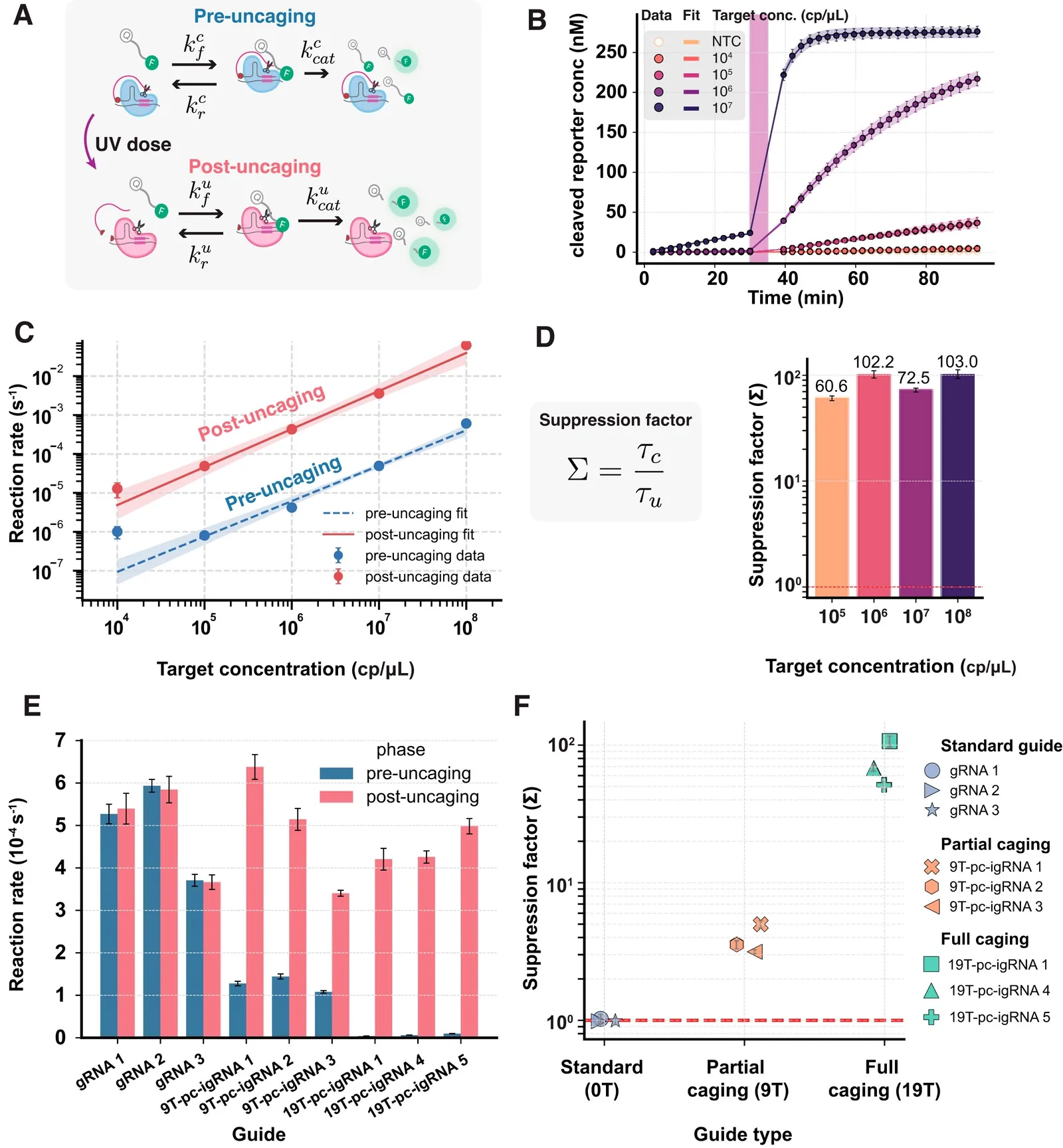
Kinetics of LUCas and suppression factor measurements. (**A**) Schematic of LbuCas13a *trans*-cleavage kinetics using LUCas guides. (**B**) Cleaved reporter concentration as a function of time for increasing target concentrations using the 19T-pc-igRNA 1. Symbols represent experimental measurements (mean across replicates, error bars: one standard deviation), and solid lines indicate fits to a two-phase Michaelis–Menten kinetics model. Shaded regions denote the 95% confidence interval (CI) of the fitted model. (**C**) Reaction rates extracted from the two-phase fits for the pre- and post-uncaging phases. Lines represent weighted linear regressions of the form $\mathrm{Rate} = (k_{\mathrm{cat}}/K_M)[E]$, where *[E]* is the target concentration and $k_{\mathrm{cat}}/K_M$ is the apparent catalytic efficiency. Shaded regions indicate the 95% CI of the fitted regression. (**D**) Suppression factor, defined as the ratio of post- to pre-uncaging reaction rates, plotted as a function of target concentration. (**E**) Pre- and post-uncaging reaction rates for guides containing increasing lengths of the interfering DNA segment (0T, 9T, 19T). (**F**) Corresponding suppression factors for guides with increasing interfering segment length. For panels (C–F), error bars represent standard error, obtained by combining variability across experimental replicates and uncertainty from the fitted rates in quadrature.

As demonstrated in the previous section, the LUCas system allows the *trans*-cleavage activity of Cas13a enzyme to be rapidly switched from a caged to a photo-uncaged, active state using a brief light pulse. We next characterized the enzyme kinetics in these two distinct states, a prerequisite for exploring the design space of LUCas guides and enabling quantitative assays. We first conducted a sweep across 5 orders of magnitude in target concentration and measured the dynamics of cleaved reporter concentration during a pre-uncaging phase followed by a post-uncaging phase, which was triggered by a 60-s UV-light exposure (UV intensity: 16.1 mW/cm$^{2}$, corresponding to full or saturating photo-uncaging; see Fig. 1C). For regular guide-target activation, the *trans*-cleavage activity has been shown to be well-approximated by a Michaelis–Menten enzyme kinetics model [30]. Here, for the LUCas system, we hypothesized that the cleaved reporter dynamics could be predicted by a biphasic Michaelis–Menten enzyme kinetics model, with distinct kinetic parameters for the caged and uncaged RNP states. In the absence of illumination, the measured pre-uncaging kinetics report the caged or suppressed state, while after saturating illumination, the post-uncaging kinetics report the uncaged or active state. This is given by the following equations for the caged and uncaged RNP states, respectively (Fig. 2A):


\begin{eqnarray*}
E^c + S \mathrel {\mathop {\rightleftharpoons }^{\, k_f^c}_{\, k_r^c}} E^cS \xrightarrow {k_{\mathrm{cat}}^c} E^c + P,
\end{eqnarray*}

\begin{eqnarray*}
E^u + S \mathrel {\mathop {\rightleftharpoons }^{\, k_f^u}_{\, k_r^u}} E^uS \xrightarrow {k_{\mathrm{cat}}^u} E^u + P,
\end{eqnarray*}

where *E* is the target-activated RNP complex, *S* is the substrate of uncleaved reporter molecules, *ES* is the RNP–reporter complex, and *P* is the cleaved reporter whose fluorescence we measure. The kinetic parameters $k_f$ and $k_r$ are the forward and reverse rates of *ES* formation, and $k_{\mathrm{cat}}$ is the catalytic turnover rate. Superscripts *c* and *u* denote the caged and uncaged RNP states, respectively.

Under the assumptions of $[S] \ll K_{M}$, where $K_{M} = (k_{r} + k_{\mathrm{cat}})/k_{f}$ is the Michaelis–Menten constant, one can simplify the kinetics and write a closed-form solution for the cleaved reporter concentration. This assumption holds in our system since $[S] \approx 400~\mathrm{nM}$ and $K_{M} \sim \mathcal {O}(\mu \mathrm{M})$ (from previous measurements, see for instance [30]). This gives:


\begin{eqnarray*}
[P](t) = S_{0}\, \bigl [1 - \exp (-t/\tau )\bigr ],
\end{eqnarray*}

where $S_{0}$ is the initial uncleaved reporter concentration and $\tau = K_{M}/(k_{\mathrm{cat}} E_{0})$ is the characteristic reaction time scale. Here $S_0$ is the initial reporter concentration and $E_0$ the initial concentration of target-activated RNP.

Applying this model to the pre-uncaging phase, which reports the caged RNP state, one obtains:


\begin{eqnarray*}
[P](t) = S_{0}\, \bigl [1 - \exp (-t/\tau _c)\bigr ],
\end{eqnarray*}

where $\tau _c = K_M^c/(k_{\mathrm{cat}}^c E_{0})$.

For the post-uncaging phase, which after $t = t_{\mathrm{stim}}$ reports the uncaged RNP state, we write:


\begin{eqnarray*}
[P](t^{\prime }) = S_0 - \bigl (S_{0} - P_{\mathrm{stim}}\bigr )\, \exp (-t^{\prime }/\tau _u),
\end{eqnarray*}

where $t^{\prime } = t - t_{\mathrm{stim}}$, $\tau _u = K_M^u/(k_{\mathrm{cat}}^u E_{0})$, and $P_{\mathrm{stim}}$ is the cleaved reporter concentration at the stimulation time $t_{\mathrm{stim}}$.

We performed non-linear least-squares fitting of the pre- and post-uncaging data and observed excellent agreement with this biphasic Michaelis–Menten model (Fig. 2B). From the fits, we extracted the characteristic reaction rates $1/\tau _c$ and $1/\tau _u$ as functions of target concentration. Over 4 decades in target concentration, both caged-state and uncaged-state reaction rates exhibited a linear dependence on target concentration ($R^{2}=0.99$ for the caged state and $R^{2}=0.95$ for the uncaged state) (Fig. 2C). Thus, as predicted by the Michaelis-Menten model, the reaction rates satisfy $1/\tau _c = (k_{\mathrm{cat}}^c/K_M^c)\, E_{0}$ and $1/\tau _u = (k_{\mathrm{cat}}^u/K_M^u)\, E_{0}$.

From linear fits of reaction rate versus target concentration, we estimate


\begin{eqnarray*}
\frac{k_{\mathrm{cat}}^c}{K_M^c} = 3.4\times 10^{6}~\mathrm{M^{-1}\, s^{-1}} \quad \mathrm{[95\%\ CI:\ }2.2\mathrm{-}5.3\times 10^{6}\mathrm{]},
\end{eqnarray*}

and


\begin{eqnarray*}
\frac{k_{\mathrm{cat}}^u}{K_M^u} = 3.3\times 10^{8}~\mathrm{M^{-1}\, s^{-1}} \quad \mathrm{[95\%\ CI:\ }1.4\mathrm{-}8.3\times 10^{8}\mathrm{]}.
\end{eqnarray*}

We define the suppression factor (*Σ*), as the ratio of uncaged to caged enzyme catalytic efficiency,


\begin{align*}
\Sigma = \frac{(k_{\mathrm{cat}}^u/K_M^u)}{ (k_{\mathrm{cat}}^c/K_M^c)}.
\end{align*}

Equivalently, this can be written as the ratio of the characteristic reaction time scales, $\Sigma = \tau _c/\tau _u$. For the 19T-pc-igRNA, we measure $\Sigma \approx 100$, corresponding to an approximately hundred-fold increase in activity upon illumination (Fig. 2D). Notably, *Σ* is largely independent of target RNA concentration (Fig. 2D), indicating that suppression and photo-uncaging are governed by guide caging rather than guide–target binding kinetics.

To confirm this independence, we next measured the pre- and post-uncaging reaction rates across a range of different guide-target combinations and for two different lengths of the interfering DNA segment, one consisting of 9 and the other consisting of 19 thymine (T) bases. We selected 9T- and 19T-pc-igRNAs as representative partial- and full-caging designs, respectively: 9T guides retain measurable pre-uncaging activity, whereas 19T guides produce strong suppression before photo-uncaging. Together with standard guides lacking an interfering segment, these guides span the functional regimes needed for this study: no caging, partial caging, and full caging.

As expected, canonical guides with no interfering DNA segment or photocleavable linker showed the same reaction rates pre- and post-uncaging, and suppression factors $ \Sigma \approx$ 1 (Fig. 2E). Partially interfering guides (9T-pc-igRNAs) allowed the enzyme to retain some activity before uncaging and recover full activity after photo-uncaging, whereas fully interfering guides (19T-pc-igRNAs) showed the strongest suppression before photo-uncaging. Importantly, while absolute reaction rates varied across different guide–target combinations, the measured suppression factors clustered into logarithmically separated regimes. These correspond to $\Sigma \approx 1$ (no suppression for standard guides), $\Sigma \approx 3$–5 (partial suppression for 9T-pc-igRNAs), and $\Sigma \approx 50$–125 (full suppression for 19T-pc-igRNAs) (Fig. 2F). These results demonstrate that the photo-uncaging and associated suppression factors are a strong function of the interfering DNA length and are relatively insensitive to the guide-target combination, demonstrating a direct light-based control of the *trans*-cleavage activity of the enzyme.

### Suppression of non-specific enzyme activity and limit-of-detection measurements using LUCas

In the previous section, we demonstrated that caging and uncaging of LUCas guides, in the presence of target RNA, could be characterized via a suppression factor based on the kinetic rates. We next characterized the impact of RNP caging and uncaging on the non-target-dependent background activity of the enzyme. This analysis leads to and culminates in our ability to predict and measure the limit-of-detection of a Cas13a reaction using a single guide with the LUCas system.

**Figure 3.**
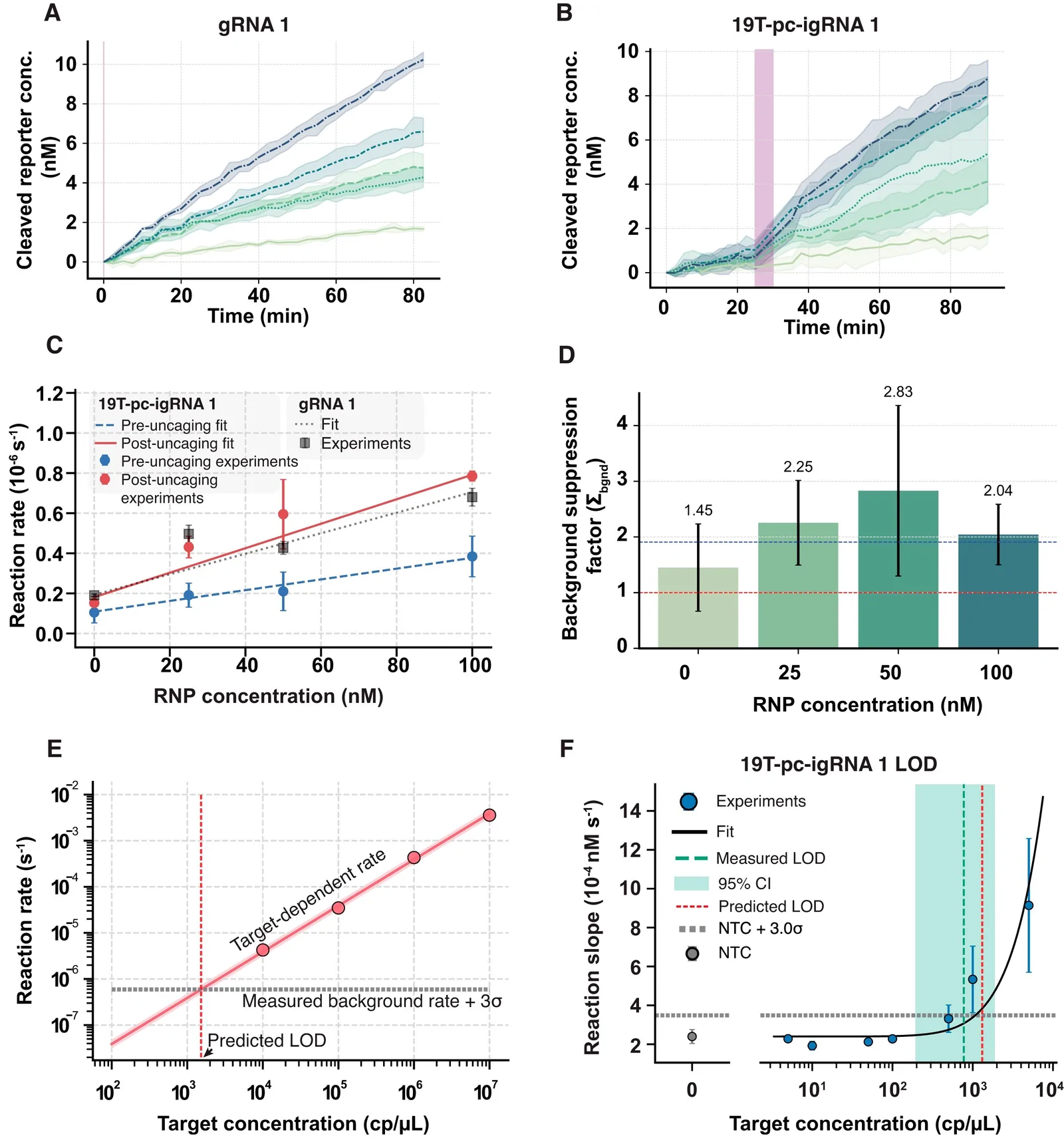
Background kinetics of LUCas and limit-of-detection measurements. (**A, B**) Cleaved reporter concentration as a function of time showing background *trans*-cleavage activity of the RNP with standard guides and pc-igRNAs, respectively, in the absence of target RNA. Solid lines indicate the mean across experimental replicates, and shaded regions denote the 95% confidence interval (CI). (**C**) Reaction rates extracted from linear fits to the pre- and post-uncaging phases of the curves shown in panels (A, B), enabling quantification of background activity. (**D**) Background suppression factor, defined as the ratio of post- to pre-uncaging reaction rates in the absence of target RNA, demonstrating that LUCas guides suppress background activity by approximately two-fold. (**E**) Post-uncaging reaction rate as a function of target concentration (symbols with linear fit), shown together with the detection threshold derived from background rates measured at 25 nM RNP (mean $+\, 3\times$ SD; horizontal dashed line). Mapping this threshold onto the fit yields the predicted limit-of-detection (LOD) (vertical dashed line). (**F**) Experimental LOD measurements for the 19T-pc-igRNA 1 guide. Post-uncaging reaction rates were fitted with a three-parameter logistic model (solid curve). The experimentally determined LOD is indicated by the median estimate (vertical dashed line) with its 95% CI (shaded band), shown alongside the predicted LOD (vertical dotted line). For panels (C, D, F), error bars represent the standard error, obtained by combining variability across experimental replicates and uncertainty from the fitted rates in quadrature.

To characterize background rates, we measured the dynamics of the cleaved reporter concentration over a range of RNP concentrations. For standard guides, we observed a steady linear increase in cleaved reporter concentration with time, at a rate that is RNP-dependent and clearly greater than the no-RNP control (Fig. 3A). This no-RNP rate represents the noise floor of our measurements and results from non-enzyme-related background processes that may include reporter degradation [32]. In contrast, for the 19T-pc-igRNA, we detected suppressed background activity in the pre-uncaging phase, followed by an increased rate after uncaging (Fig. 3B). Importantly, this rate change after uncaging occurred only when RNP was present, which rules out a light-induced increase in reporter degradation as the primary explanation for this change. Reaction rates extracted from least-squares fits to the cleaved reporter concentration data showed linear scaling with RNP concentration for both pre- and post-uncaging phases, with the post-uncaging background rate being comparable to the standard guide case, as expected (Fig. 3C). We can therefore model the RNP-dependent background reaction rate as:


\begin{eqnarray*}
\frac{1}{\tau _{\mathrm{bgnd}}} = \left(\frac{k_{\mathrm{cat}}}{K_{M}}\right)_{\mathrm{bgnd}} [\mathrm{RNP}],
\end{eqnarray*}

where the background catalytic efficiencies are found to be


\begin{eqnarray*}
\left(\frac{k_{\mathrm{cat}}}{K_{M}}\right)_{\mathrm{bgnd}}^{c} = 2.7~\mathrm{M^{-1}\, s^{-1}} \quad \mathrm{[95\%\ CI:\ }1.2\mathrm{-}4.1\mathrm{]},
\end{eqnarray*}

and


\begin{eqnarray*}
\left(\frac{k_{\mathrm{cat}}}{K_{M}}\right)_{\mathrm{bgnd}}^{u} = 6~\mathrm{M^{-1}\, s^{-1}} \quad \mathrm{[95\%\ CI:\ }3.2\mathrm{-}8.9\mathrm{]},
\end{eqnarray*}

where the superscripts *c* and *u* correspond to the caged and uncaged states of the RNP, respectively, and the subscript *bgnd* refers to this being the background, no-target parameter. Thus, the LUCas guides suppress Cas13a RNP background activity by roughly two-fold in the caged state, again in a manner that is independent of RNP concentration (Fig. 3D).

Using these measurements of background activity, we next established the limit-of-detection (LOD) of the LUCas system. Since our analysis is rate-based, we defined the detection threshold as


\begin{eqnarray*}
LOD_{\mathrm{signal}} = \mu _{\mathrm{NTC}} + 3\sigma _{\mathrm{NTC}},
\end{eqnarray*}

where $\mu _{\mathrm{NTC}}$ and $\sigma _{\mathrm{NTC}}$ are the mean and standard deviation of the reaction rate for the non-target control (NTC), computed across three replicate wells. The LOD in signal space is reached when the target-dependent post-uncaging rate equals this threshold, i.e. $1/\tau _u = LOD_{\mathrm{signal}}$. This implies that the LOD in concentration space is given by


\begin{eqnarray*}
LOD_{\mathrm{target\,\,conc}} = \bigl (\mu _{\mathrm{NTC}} + 3\sigma _{\mathrm{NTC}}\bigr ) \left(\frac{K_{M}}{k_{\mathrm{cat}}}\right)^{u}.
\end{eqnarray*}

Using the above model and our measured target-dependent and background kinetic parameters, we predict a limit of detection (LOD) of $1500~\mathrm{copies}/\mu \mathrm{L}$ (95% CI: 1250–1800) for a single reaction (Fig. 3E). We next determined the LOD experimentally by measuring post-uncaging reaction slopes across increasing target concentrations for the 19T-pc-igRNA 1. The slope data were fit using a three-parameter sigmoid model [33], and the same NTC-based LOD threshold was applied (Fig. 3F). Mapping the resulting threshold from reaction-rate space to concentration space yielded an experimental LOD of $815~\mathrm{copies}/\mu \mathrm{L}$ (95% CI: 200–1881). Notably, the predicted and experimentally measured LOD values are in agreement, as evidenced by their overlapping confidence intervals, indicating that the LOD of the current LUCas system is set by the background catalytic activity of the enzyme (Fig. 3F). This conclusion is further supported by measurements of two additional guide–target combinations using the same 19T interfering segment, both of which yielded comparable LOD values of $430~\mathrm{copies}/\mu \mathrm{L}$ (95% CI: 250–900) and $520~\mathrm{copies}/\mu \mathrm{L}$ (95% CI: 12–730) (Supplementary Fig. S8).

### Mechanism of suppression and photo-uncaging in pc-igRNAs

**Figure 4.**
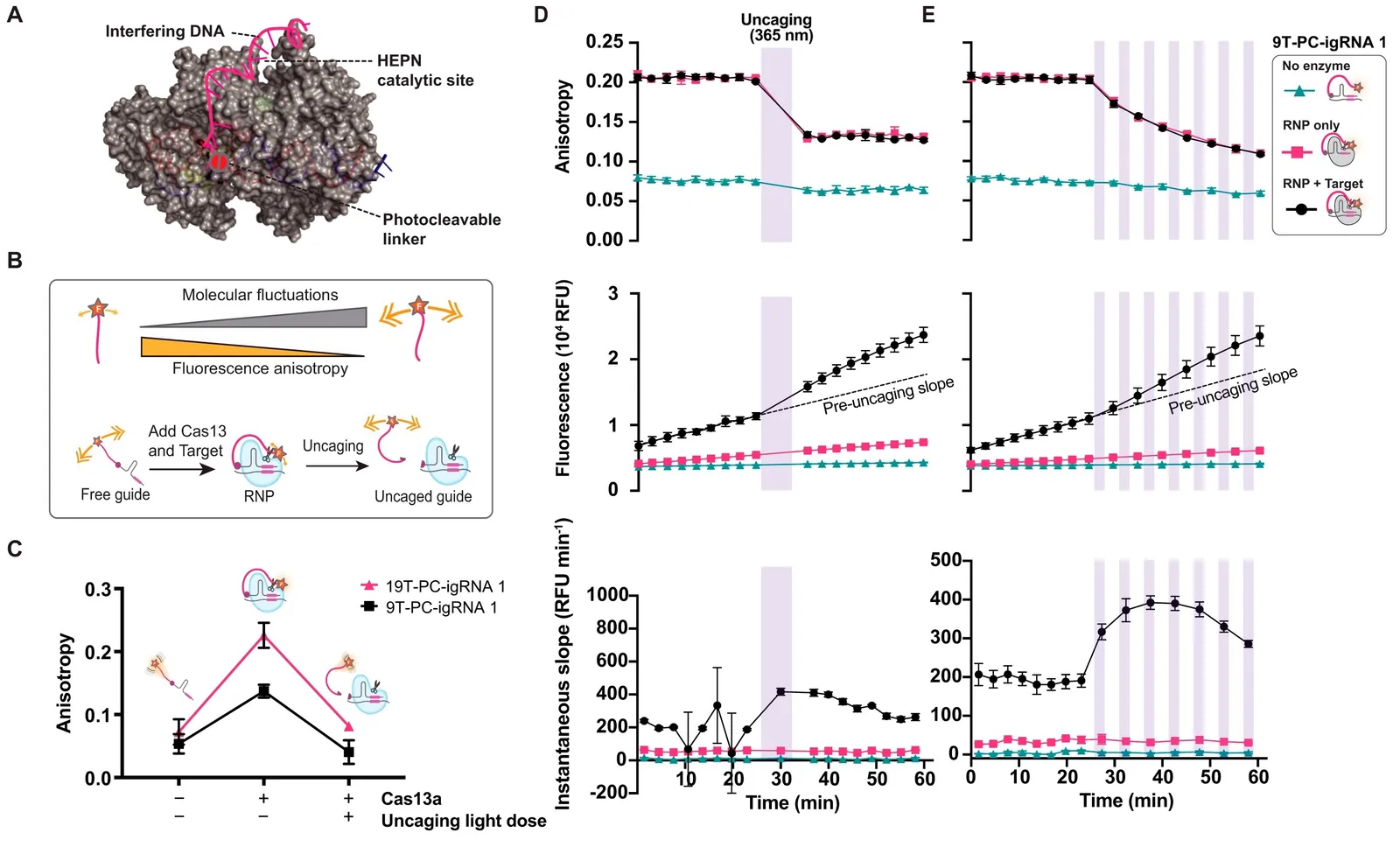
Mechanism of photo-uncaging using photocleavable interfering guide RNAs. (**A**) Structure of the LbuCas13a:19T-pc-igRNA:target RNA complex. (**B**) Schematic representation of the fluorescence anisotropy measurements used to elucidate the mechanism for LUCas. (**C**) Fluorescence anisotropy measurements using 5′ fluorescently labeled gRNA for different lengths of the interfering DNA segment. Freely suspended CRISPR guides (gRNAs) lead to a low fluorescence anisotropy value, while gRNAs complexed with LbuCas13a, which forms a ribonucleoprotein (RNP), lead to a higher anisotropy value. Upon UV illumination, the anisotropy relaxes to a lower value when compared to the RNP case. This observation is consistent with photo-release of the interfering ssDNA segment from the RNP complex. Data points show mean ± standard deviation (SD). (**D**) Simultaneous temporal measurements of fluorescence anisotropy and LbuCas13a *trans*-cleavage activity (via cleaved reporter intensity) show the photo-uncaging of LbuCas13a by a single UV dosage (365 nm). The change in *trans*-cleavage rate occurs concurrently with the change in fluorescence anisotropy. (**E**) Simultaneous temporal measurements of fluorescence anisotropy and LbuCas13a *trans*-cleavage activity show that the photo-uncaging of LbuCas13a can be precisely tuned by pulsed UV illumination. Each UV pulse photoactivates a new population of LbuCas13a, as confirmed by a concurrent change in anisotropy. In panels (D and E), error bars represent the standard error of the mean across three replicates.

The LUCas system leverages pc-igRNAs, which are hypothesized to work by blocking RNA cleavage through the *trans*-cleavage catalytic domain on Cas13a [31]. Molecular dynamics (MD) simulations predict that the ssDNA segment attached to the 5′ end of the guide is held close to the Cas13a surface in a manner that colocalizes the ssDNA segment with the catalytic HEPN domain (Fig. 4A). Since these simulations, by themselves, do not establish a high-resolution binding structure [34], fluorescence anisotropy measurements were used to assess the molecular rotational rate of the partially blocking 9T-pc-igRNA, alongside simultaneous activity measurements to test two experimentally accessible predictions: that pc-igRNAs still form stable RNP complexes, and that photo-uncaging releases the interfering DNA segment while restoring *trans*-cleavage activity (Fig. 4B).

Fluorescence anisotropy has been a powerful tool for quantifying protein binding, protein denaturation, and protein dynamics, where it provides quantitative information about rotational degrees of freedom [35]. More recently, this technique has been used to study gRNA-target RNA binding affinities [36]. Small molecules such as gRNA that are free in solution have rapid rotational rates and hence low anisotropy values, while molecules bound to larger proteins have slower rotational rates, leading to higher anisotropy values [35]. Fluorescence anisotropy of a 5′ fluorescently labeled 9T-pc-igRNA was measured before and after UV uncaging, and the *trans*-cleavage activity was simultaneously monitored to establish a mechanism for LUCas.

For the 19T-pc-igRNA without Cas13a, we measured low anisotropy values corresponding to the rotational rate of free molecules (Fig. 4C). Once Cas13a and the target RNA are added, we found a significant increase in anisotropy, indicating a stable RNP complex formation (Fig. 4C). Using two versions of pc-igRNAs (one with a 9T and the other with a 19T interfering segment), we found that the increase in anisotropy is greater for the longer guide (19T) than the shorter one (230% for 19T versus 157% for 9T). Using the average size of 0.5 nm per nucleotide and the total number of nucleotides on the interfering strand, the total length is estimated to be 45 Å for the 9T and 95 Å for the 19T [37]. These data are consistent with a picture in which the longer DNA segment spans the distance between the maturation site and HEPN domains on Cas13a (distance *≈* 50 Å) and, hence, more effectively interferes with reporter binding and catalytic activity, consistent with increased suppression by these guides (Figs 4A, 2E and F). Upon subsequent UV exposure, we found that the measured anisotropy fell back to values similar to those of free guide in solution, indicating efficient photocleavage and release of the interfering DNA segment from Cas13a (Fig. 4C).

How do these changes in anisotropy correlate with the *trans*-cleavage activity of Cas13a? To understand this correlation, fluorescence anisotropy and *trans*-cleavage activity were measured simultaneously in the same experiment. We found that a single exposure of UV light (duration: 40 s, UV dose: 160 *μ*J) led to a step decrease in fluorescence anisotropy for 9T-pc-igRNA with Cas13a, independent of the presence of target RNA. In contrast, no change in anisotropy was observed for the case of free guides, even in the presence of target RNA (Fig. 4D). This step decrease in anisotropy temporally corresponded with a step increase in *trans*-cleavage activity (Fig. 4D).

When exposed to several shorter exposures of UV light (duration: 10 s, UV dose: 40 *μ*J), we observed rapid, step decreases in fluorescence anisotropy that corresponded with step increases in *trans*-cleavage activity (Fig. 4E), consistent with additional populations of the RNP being photo-activated. The response eventually saturates due to the consumption of uncleaved reporter molecules in solution (Fig. 4E, bottom).

Our results from these fluorescence anisotropy measurements indicate that pc-igRNAs effectively form RNPs despite the presence of the interfering DNA segment at the 5′ end of the guide. Furthermore, recovery of *trans*-cleavage activity after UV uncaging is accompanied by a rapid decrease in anisotropy, consistent with photocleavage and release of the interfering DNA segment from the Cas13a RNP. This release process is faster than the timescale of our measurements of both reporter cleavage and anisotropy, as we did not capture the transition from off to on.

These data support a model in which the attached interfering DNA segment suppresses Cas13a catalytic output while preserving target-bound RNP formation, and light-triggered cleavage relieves this suppression. Overall these experiments support the HEPN-proximal inhibition model and motivate future structural studies of the pc-igRNA-bound complex.

### Temporal barcoding with the LUCas system

Pathogen co-infections in a single host are common in nature and in clinical settings [38–40]. For that reason, multiplexing is a desired feature in most diagnostic assays. For CRISPR-based diagnostics, this is possible using different gRNAs that target distinct pathogens or viruses. However, an outstanding issue is distinguishing the activity from different guides targeting different pathogens in a single bulk reaction, since modest sequence dependence of the *trans*-cleavage activity limits assays to using a single channel for reporter fluorescence.

**Figure 5.**
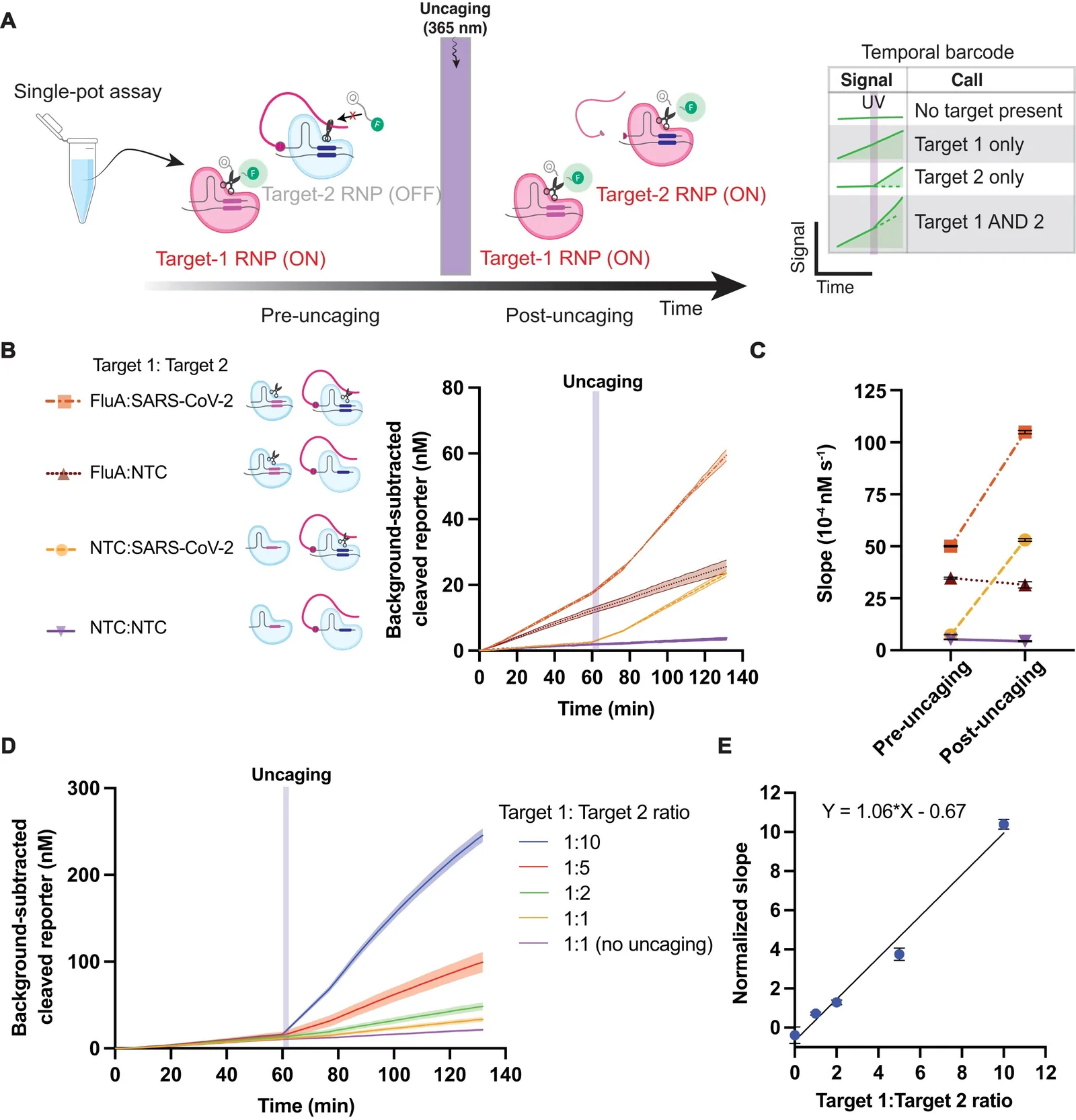
LUCas enables temporal barcoding assays for detecting multiple targets in a single reaction. (**A**) Schematic representation of the temporal barcoding assay, which enables differentiation between the *trans*-cleavage activity of one standard guide RNA (gRNA) and one photocleavable guide (pc-igRNA) in a single CRISPR reaction. (**B**) Demonstration of the LUCas assay for detecting two viruses (FluA and SARS-CoV-2) in a single reaction using a temporal-barcoding approach. The standard guide targets FluA (gRNA FluA), while the photocleavable guide is complementary to a segment of the SARS-CoV-2 virus genome. The curves show the mean of three replicates, and the shaded regions indicate the SEM. (**C**) The distinct temporal barcodes, based on measured rate changes (pre-stim and post-stim), allow distinguishing between the four possible outcomes in a single reaction. Error bars represent the mean ± SEM (*n* = 3). (**D**) Measured cleaved reporter concentration over time of a reaction with a mixture of different target concentration ratios. Error bars represent the SEM (*n* = 3). (**E**) Scatter plot of normalized slope difference $(R^{\prime }_{post-stim} - R^{\prime }_{pre-stim})/R^{\prime }_{pre-stim}$, where $R^{\prime }$ is the reaction slope measured using a linear fit, and ratio of target concentrations shows a linear relationship, as expected. This relationship can be used to quantitatively estimate the target concentration ratios of a sample, given knowledge about individual guide-dependent activity. Data points show the mean ± SEM (*n* = 3).

In earlier work, we demonstrated one type of multiplexing strategy by leveraging the distinct kinetic signatures of different guides and interfering guide lengths, which we referred to as “kinetic barcoding” [31]. Here, we leveraged the LUCas system to introduce an alternative multiplexing strategy that we call “temporal barcoding.” This strategy enables separately identifying the *trans*-cleavage activity of two or more different guides (e.g. one standard gRNA and one 19T-pc-igRNA) in a single CRISPR reaction using light to temporally control when a reaction is activated (Fig. 5A). Initially, in the simplest case of two different target RNAs of interest, only the RNP with the standard guide is active and the RNP with the 19T-pc-igRNA is suppressed. After a fixed duration of time, the reaction is exposed to UV light, activating the RNP with the 19T-pc-igRNA. This will result in a unique change in fluorescence signal over time, or a temporal barcode, that depends on the presence or absence of the two targets.

To demonstrate this method, we combined a mixture of Influenza A (FluA) and SARS-CoV-2 whole-genome RNA in one CRISPR reaction using a standard gRNA for FluA and a 19T-pc-igRNA for SARS-CoV-2. The measured fluorescence intensity and slope (before and after UV illumination) are shown in Fig. 5B and C, respectively. We find that reaction slopes pre- and post-UV can be temporally segmented based on the presence of one or more targets (Fig. 5C). This demonstration shows how temporally separating the activity of two guides in a single reaction enables multiplexed detection of two targets using LUCas.

To test the practically relevant case where the concentrations of the two targets are different, we varied the 19T-pc-igRNA target concentration and measured the cleaved reporter concentration (Fig. 5D) along with the pre-stim and post-stim reaction rates (Fig. 5E). The post-uncaging reaction slopes showed an increase that was dependent on target concentration ratio (Fig. 5E). In addition, the normalized slope difference $(R^{\prime }_{post-stim} - R^{\prime }_{pre-stim})/R^{\prime }_{pre-stim}$, where $R^{\prime }$ is the reaction slope, showed a linear relationship with the ratio of target concentrations, as expected (Fig. 5E). Furthermore, the slope of the linear fit was found to be proportional to the ratio of activities of the two guides for a given target concentration. This implies that one can quantitatively estimate the concentrations of at least two different targets in a single CRISPR reaction using this technique.

### Performance for clinical applications

**Figure 6.**
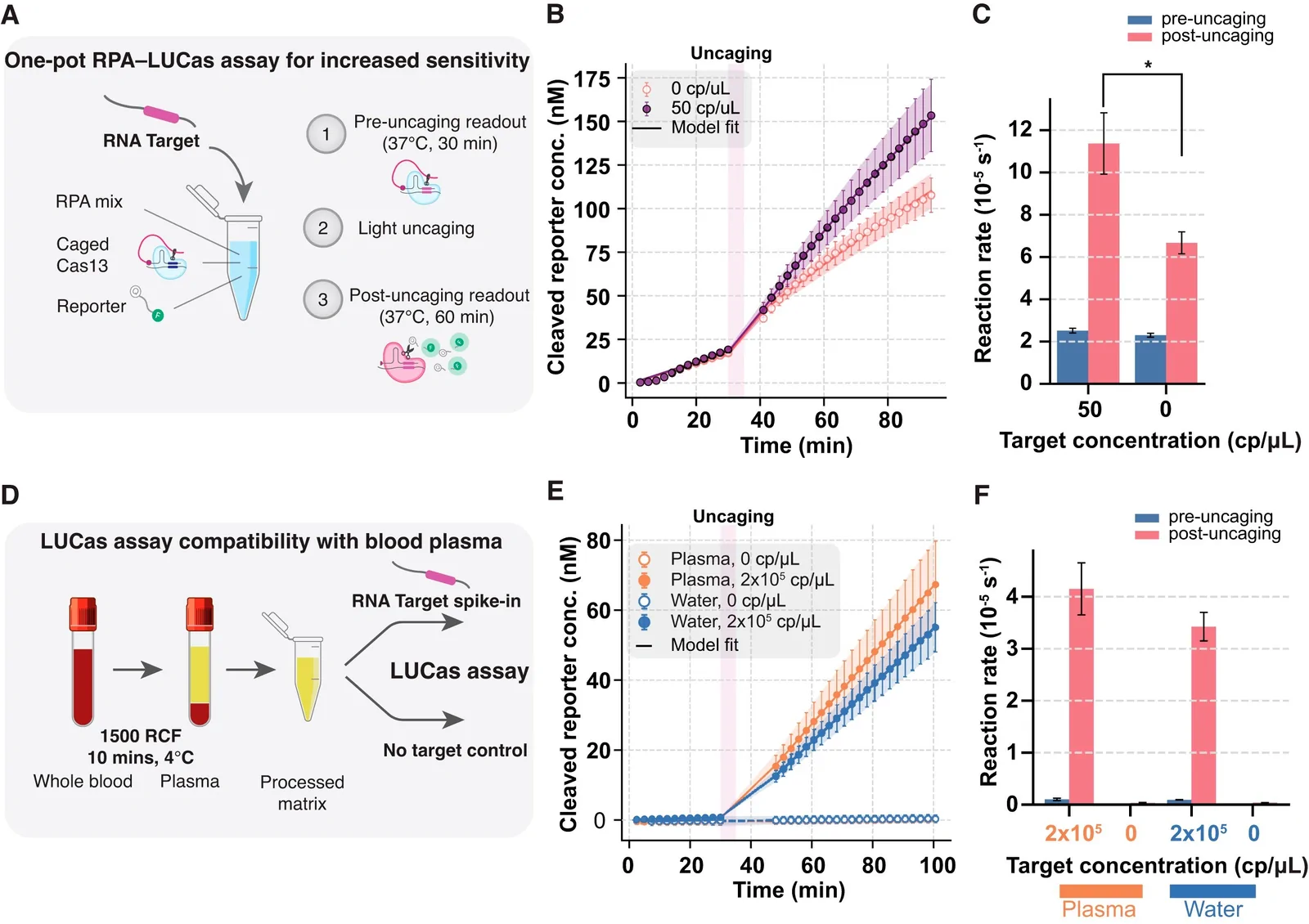
LUCas enables one-pot RPA–Cas13a detection and remains active in target-spiked plasma. (**A**) Schematic of the one-pot RPA–LUCas assay. Target RNA, RPA reagents, photocaged Cas13a RNP, and fluorescent reporter are combined in a single reaction. Reporter cleavage is monitored before and after photo-uncaging to quantify caged and photo-uncaged Cas13a activity. (**B**) Cleaved reporter concentration as a function of time for target-containing reactions (50 cp/*μ*l) and non-target control (0 cp/*μ*l). Symbols represent experimental measurements and solid lines indicate fits to a biphasic Michaelis–Menten kinetics model (error bars: one standard deviation and shaded region: 95% CI of the fit). (**C**) Reaction rates extracted from model fits to the pre- and post-uncaging phases shown in panel (B). (**D**) Schematic of the target-spiked blood plasma workflow used to assess LUCas performance in a complex sample matrix. Plasma was separated from whole blood, processed, and either spiked with target RNA or retained as a no-target control prior to LUCas detection. (**E**) Cleaved reporter concentration as a function of time for target-spiked reactions in water and processed plasma. LUCas maintains low pre-uncaging activity and robust light-activated reporter cleavage in both matrices. Symbols represent experimental measurements and solid lines indicate fits to the biphasic Michaelis–Menten kinetics model. (**F**) Reaction rates extracted from model fits to the pre- and post-uncaging phases shown in panel (E), demonstrating preserved target-dependent photo-uncaging in processed plasma. In panels (C and F) error bars represent the standard error, obtained by combining variability across replicate wells and uncertainty from the fitted rates in quadrature.

To evaluate the LUCas system for clinically relevant applications, two key factors were considered: achieving high sensitivity and compatibility with complex clinical matrices. To address the former, LUCas was coupled with recombinase polymerase amplification (RPA), an isothermal amplification technique that enables sensitive nucleic acid detection [41]. An overview of the LUCas-RPA assay is shown in Fig. 6A. Using this approach, LUCas successfully detected 50 cp/*μ*l of synthetic SARS-CoV-2 RNA whole virus (Fig. 6B and C). To assess matrix compatibility, a synthetic target was spiked into a blood plasma sample treated with Proteinase K (Fig. 6D), and the resulting fluorescence kinetics were compared to those obtained in nuclease-free water at the same target concentration (Fig. 6E). The performance of LUCas in plasma was comparable to that of the nuclease-free water control (Fig. 6F), demonstrating its compatibility with processed, target-spiked biological matrices and supporting its potential for clinical diagnostic applications.

## Discussion

In this work, we introduced the LUCas system, which allows direct light control of Cas13a *trans*-cleavage activity, independent of RNP formation and guide-target binding kinetics. LUCas allows modulation of the catalytic output of the enzyme in the caged and photo-uncaged states without perturbing the molecular recognition steps; in other words, suppression is present even when target RNA is bound to the RNP. Upon brief light exposure, the Cas13a activity rapidly rises to the full rate expected for a given guide-target combination.

We characterized the kinetics of the LUCas system and find that it follows a biphasic Michaelis–Menten model with distinct kinetic parameters for the pre- and post-uncaging phases. Importantly, this change in kinetic rates can be captured through a single, generalizable phenomenological suppression factor, which is the ratio of post-uncaging to pre-uncaging reaction rates, and it is independent of target concentration and guide-target combinations. We find that this suppression factor is *∼*100-fold for the longest interfering DNA segment tested (19T) and can be tuned by two independent control knobs, namely the interfering DNA length and light dose, respectively. In contrast, target-independent background activity is more modestly suppressed, by approximately two-fold in the caged state. Thus, LUCas strongly suppresses target-dependent trans-cleavage activity prior to uncaging while also reducing the RNP-dependent background above which a positive signal must rise to be detected. The biphasic kinetic model and the phenomenological suppression factor that we introduce could be a useful general framework to quantify how enzyme activity is modulated in other gating strategies, including alternative light-control methods.

Using our measurements of both target-dependent and background kinetic rates, we quantitatively predict the limit of detection of the LUCas system to be on the order of 1000 cp/*μ*l and find excellent agreement with experiments across three different guide-target combinations. Lastly, using the light-controlled features of the LUCas system, we introduce a multiplexing strategy that we call “temporal barcoding” and use it to detect a co-infection of SARS-CoV-2 and influenza A in a single bulk reaction, using light to sequentially activate two populations of RNPs, each one targeting a specific virus. Further, we showed that the relative abundance of two viral RNAs can be estimated in a single bulk reaction. For clinically relevant applications, we show that LUCas is compatible with isothermal amplification techniques and complex sample matrices.

In contrast to other light-control strategies for CRISPR enzymes (Supplementary Table S2), the LUCas system directly gates enzymatic *trans*-cleavage activity, with or without the target bound, rather than perturbing RNP formation or suppressing target binding. This strategy avoids the high background activity of unbound or apo-Cas13a, reduces background activity of the RNP, and allows time for all target RNA to bind to the RNP complex before the reaction starts. In terms of chemical modification, LUCas requires only a single photocleavable molecule per guide (compared to three or more in Cas12a light-activation strategies [7, 10, 11, 14]), and it does not perturb guide structure or guide–target binding kinetics, thereby simplifying assay design and alleviating complex optimization requirements. While we mostly focused on amplification-free detection in this work, we have demonstrated that LUCas is compatible with amplification steps such as RPA. In the future this combination can be explored further to more effectively quench Cas13a *trans*-cleavage activity until the final detection step and to prevent degradation of amplification substrates and products, potentially leading to improved sensitivity.

We have demonstrated LUCas for the detection of two different targets using temporal barcoding, and the system is well-positioned for development of further multiplexing strategies. In particular, different targets can be encoded by including photocleavable moieties that are cleaved at distinct wavelengths [42]. To this end, the photo-uncaging kinetics and effective reaction rate model we develop and validate here are a useful foundation and design tool (Supplementary data). LUCas can also be combined with other multiplexing strategies, including a light-triggered form of “kinetic barcoding” [31], wherein the photocleavable molecule can be placed at different locations at the 5′ end of the gRNA to enable distinct and well-characterized suppression factors for different gRNA populations aimed at detecting different targets. LUCas also lends itself to spatial barcoding of different guides. This could be implemented on patterned surfaces or beads, with the LUCas system activating Cas13a *trans*-cleavage activity where and when needed. Finally, to improve implementation accessibility, modular design strategies such as click chemistry could be used to convert regular gRNAs into pc-igRNAs [43].

A defining feature of the LUCas system is the combination of strong enzymatic suppression with rapid activation, such that Cas13a binding and catalytic output can be cleanly decoupled. Further study of this behavior will help clarify how secondary interactions between the interfering DNA segment and surface residues of Cas13a modulate accessibility of the HEPN catalytic domains. In tandem, statistical mechanics and molecular dynamics models could provide a predictive framework for how interfering segment length, flexibility, and sequence set the degree of suppression and the kinetics of activation after photocleavage. Engineering Cas13a and the pc-igRNAs to exploit these interactions more effectively has the potential to further enhance suppression, thereby improving signal-to-background ratios and expanding the performance envelope of Cas13a-based detection systems.

## Supplementary Material

gkag848_Supplemental_File

## Data Availability

All data supporting the findings of this study, including raw fluorescence time series, processed kinetic measurements, analysis scripts, and figure-generation code, are publicly available via our Dryad Digital Repository (https://datadryad.org/dataset/doi:10.5061/dryad.7wm37pw7f).
