# Supplementary material for: LUCas: Light-Uncaged Cas13a using photocleavable interfering guide RNAs
Source: Nucleic Acids Res. 2026 Aug 31;54(16):gkag848. doi: 10.1093/nar/gkag848 (PMC13527167; doi:10.1093/nar/gkag848)
Supplement: Supplementary file 1 [file gkag848_supplemental_file.pdf]

Supplementary Information

LUCas: Light-Uncaged Cas13a using photocleavable interfering guide RNAs

Carlos F. Ng†, Deepak Krishnamurthy†, Andres Dextre, Aymeric Chorlay, Melanie Ott, Daniel A. Fletcher

† Equal contribution

Contents

1 Supplementary Text **2**

1.1 Photo-uncaging model for a single caged Cas13a guide and target 2

1.2 Model for LUCas reaction kinetics with both caged and uncaged RNP contributions 4

2 Supplementary Figures **10**

List of Figures

S1 Photocleavable Linker 10
S2 Fluorescence reporter calibration curves 11
S3 LUCas assay illumination hardware 12
S4 Characterization of the Lumidox LED array 13
S5 Absence of *trans*-cleavage activity in masked wells 14
S6 Repeatability and reproducibility of LUCas guides 15
S7 Non-specific effects of short DNA fragments 16
S8 Limit of detection analysis for other pc-igRNAs 17

List of Tables

S1 Oligonucleotides used in this study 18
S2 Overview of Light-Controlled CRISPR Diagnostic Strategies 20

1 Supplementary Text

1.1 Photo-uncaging model for a single caged Cas13a guide and target

We first model the light-dependent conversion of a caged Cas13a ribonucleoprotein (RNP) complex into an uncaged complex. This section considers the experimentally relevant case of a single photocleavable cage and a single illumination wavelength used in the paper. The model is phenomenological: it seeks to describe how an applied light dose changes the fraction of RNPs in the caged and uncaged states without explicitly modeling the microscopic photochemical intermediates.

Let $E^c(t)$ and $E^u(t)$ denote the concentrations of caged and uncaged target-bound RNPs, respectively. We assume that photoconversion is irreversible, and that no RNP is degraded or lost during illumination. Therefore,

$$E^c \xrightarrow{h\nu} E^u, \tag{1}$$

and

$$E^c(t) + E^u(t) = E_0, \tag{2}$$

where E_0 is the total concentration of target-bound RNP.

We model photoconversion as a first-order process in the remaining caged population,

$$\frac{dE^c}{dt} = -k_{\text{photo}}(t)E^c(t), \tag{3}$$

$$\frac{dE^u}{dt} = k_{\text{photo}}(t)E^c(t). \tag{4}$$

Substituting Eq. 2 in Eq. 4, the uncaged population obeys

$$\frac{dE^u}{dt} = k_{\text{photo}}(t)\,(E_0 - E^u(t))\,. \tag{5}$$

We further assume that the photoconversion rate is proportional to the incident photon flux,

$$k_{\text{photo}}(t) = \alpha(\lambda) I(t;\lambda), \tag{6}$$

where $I(t;\lambda)$ is the optical power at wavelength λ and $\alpha(\lambda)$ is an effective wavelength-dependent photosensitivity. This parameter captures the probability that incident photons lead to uncaging events. It depends on the absorption properties of the photocage, the photolysis quantum yield, and geometric or unit-conversion factors. For example, one can write

$$\alpha(\lambda) = \beta\,\varepsilon(\lambda)\,\Phi_{\text{uncage}}(\lambda), \tag{7}$$

where $\varepsilon(\lambda)$ is the extinction coefficient of the photocage, $\Phi_{\text{uncage}}(\lambda)$ is the photolysis quantum yield, and β absorbs geometry and unit conversions.

Since we are mainly interested in uncaging dynamics as a function of light dose, we define the cumulative light dose energy

$$H(\lambda, T) = \int_0^T I(t;\lambda)\,dt, \tag{8}$$

where T is the total duration of the light pulse.

Using $dH/dt = I(t;\lambda)$, Eq. 5 becomes

$$\frac{dE^u}{dH} = \alpha(\lambda)\,(E_0 - E^u(H))\,. \tag{9}$$

Separating variables and integrating gives

$$-\ln\left[\frac{E_0 - E^u(H)}{E_0 - E^u(0)}\right] = \alpha(\lambda)H. \tag{10}$$

This gives an expression for the uncaged RNP concentration as a function of light dose,

$$E^u(H) = E_0 - (E_0 - E^u(0))\exp\left[-\alpha(\lambda)H\right]. \tag{11}$$

Equivalently, defining the uncaged fraction $f^u(H) = \frac{E^u(H)}{E_0}$, and the initial uncaged fraction $f_0^u = \frac{E^u(0)}{E_0}$, we obtain

$$\boxed{f^u(H) = 1 - (1 - f_0^u)\exp\left[-\alpha(\lambda)H\right]}. \tag{12}$$

In the ideal case where no uncaged RNPs are present before illumination, $f_0^u = 0$, Eq. 12 reduces to

$$\boxed{f^u(H) = 1 - \exp\left[-\alpha(\lambda)H\right]}. \tag{13}$$

Thus, at low light dose, as expected, we get a linear scaling with light dose $f^u(H) \approx \alpha(\lambda)H$ for $\alpha H \ll 1$. At high doses the uncaged fraction saturates $f^u(H) \to 1$ which happens due to depletion of the remaining caged RNP pool.

1.2 Model for LUCas reaction kinetics with both caged and uncaged RNP contributions

The photoconversion model was subsequently connected to reporter cleavage kinetics. The key point is that both caged and uncaged target-bound RNPs can contribute to reporter cleavage, but with different catalytic efficiencies. The caged population has suppressed *trans*-cleavage activity, whereas the uncaged population has fully restored activity. This model is

directly inspired by our measurements of reporter cleavage kinetics pre- and post-uncaging which leads to a phenomenological suppression factor that scales the caged and uncaged catalytic efficiencies.

For a single guide–target pair, the caged and uncaged RNPs follow

$$E^c + S \underset{k_r^c}{\overset{k_f^c}{\rightleftharpoons}} C^c \xrightarrow{k_{\text{cat}}^c} E^c + P, \tag{14}$$

$$E^u + S \underset{k_r^u}{\overset{k_f^u}{\rightleftharpoons}} C^u \xrightarrow{k_{\text{cat}}^u} E^u + P, \tag{15}$$

where S is the uncleaved reporter substrate, P is the cleaved reporter product, and C^c and C^u are the caged and uncaged enzyme–substrate complexes.

For each population, the Michaelis constants are

$$K_M^c = \frac{k_r^c + k_{\text{cat}}^c}{k_f^c}, \qquad K_M^u = \frac{k_r^u + k_{\text{cat}}^u}{k_f^u}. \tag{16}$$

Under the quasi-steady-state approximation, the total product formation rate is

$$\frac{dP}{dt} = \frac{k_{\text{cat}}^c E_{\text{tot}}^c S}{K_M^c + S} + \frac{k_{\text{cat}}^u E_{\text{tot}}^u S}{K_M^u + S}, \tag{17}$$

where E_{tot}^c and E_{tot}^u denote the total caged and uncaged target-bound RNP concentrations in a given illumination state.

For the LUCas reporter cleavage measurements, we work in the low-substrate regime,

$$S \ll K_M^c, \qquad S \ll K_M^u. \tag{18}$$

In this limit, Eq. 17 becomes

$$\frac{dP}{dt} \approx \left[\frac{k_{\text{cat}}^c}{K_M^c} E_{\text{tot}}^c + \frac{k_{\text{cat}}^u}{K_M^u} E_{\text{tot}}^u \right] S. \tag{19}$$

Assuming reporter conservation,

$$S_0 = S + P, \tag{20}$$

we substitute $S = S_0 - P$ to obtain

$$\frac{dP}{dt} = R(H)\left(S_0 - P\right), \tag{21}$$

where the effective pseudo-first-order cleavage rate is

$$R(H) = \frac{k^c_{\text{cat}}}{K^c_M} E^c_{\text{tot}}(H) + \frac{k^u_{\text{cat}}}{K^u_M} E^u_{\text{tot}}(H). \tag{22}$$

Using

$$E^u_{\text{tot}}(H) = f^u(H) E_0, \qquad E^c_{\text{tot}}(H) = \left[1 - f^u(H)\right] E_0, \tag{23}$$

we can write

$$R(H) = E_0 \left[\frac{k^u_{\text{cat}}}{K^u_M} f^u(H) + \frac{k^c_{\text{cat}}}{K^c_M} \left(1 - f^u(H)\right) \right]. \tag{24}$$

Here we define the phenomenological suppression factor indicated by our measurements

$$\Sigma = \frac{k^u_{\text{cat}}/K^u_M}{k^c_{\text{cat}}/K^c_M}. \tag{25}$$

Thus,

$$\frac{k^c_{\text{cat}}}{K^c_M} = \frac{1}{\Sigma} \frac{k^u_{\text{cat}}}{K^u_M}. \tag{26}$$

Substituting into Eq. 24 gives

$$\boxed{R(H) = \frac{k^u_{\text{cat}}}{K^u_M} E_0 \left[f^u(H) + \frac{1 - f^u(H)}{\Sigma} \right]}. \tag{27}$$

Using the single-cage photoconversion result from Eq. 12,

$$R(H) = \frac{k^u_{\text{cat}}}{K^u_M} E_0 \left[1 - \left(1 - \frac{1}{\Sigma}\right) (1 - f^u_0) \exp\left[-\alpha(\lambda) H\right] \right]. \tag{28}$$

If the initial uncaged fraction is negligible, $f^u_0 = 0$, this reduces to

$$\boxed{R(H) = \frac{k^u_{\text{cat}}}{K^u_M} E_0 \left[1 - \left(1 - \frac{1}{\Sigma}\right) \exp\left[-\alpha(\lambda) H\right] \right]}. \tag{29}$$

Eq. 29 is the key equation which connects the instantaneous rate of reporter cleavage to the light dose via the suppression-factor weighted model for the relative contributions of the caged and uncaged RNP fractions.

Eq. 29 has two useful limits. Before illumination, $H = 0$, giving

$$R_{\text{pre}} = \frac{1}{\Sigma} \frac{k^u_{\text{cat}}}{K^u_M} E_0 = \frac{k^c_{\text{cat}}}{K^c_M} E_0. \tag{30}$$

After saturating illumination, $H \to \infty$, giving

$$R_{\text{post}} = \frac{k^u_{\text{cat}}}{K^u_M} E_0. \tag{31}$$

Therefore,

$$\frac{R_{\text{post}}}{R_{\text{pre}}} = \Sigma. \tag{32}$$

Finally, for a fixed illumination state H, Eq. 21 has the solution

$$\boxed{P(t; H) = S_0 \left[1 - \exp\left(-R(H)t\right)\right]}. \tag{33}$$

For a two-phase experiment with a pre-uncaging phase followed by a post-uncaging phase at time t_{stim}, the pre-uncaging product concentration is

$$P_{\text{pre}}(t) = S_0 \left[1 - \exp\left(-R_{\text{pre}} t\right)\right], \qquad 0 \le t < t_{\text{stim}}. \tag{34}$$

Let $P_{\text{stim}} = P_{\text{pre}}(t_{\text{stim}})$ be the cleaved reporter concentration at the time of illumination. After uncaging, with $t' = t - t_{\text{stim}}$, the product concentration is

$$P_{\text{post}}(t') = P_{\text{stim}} + (S_0 - P_{\text{stim}}) \left[1 - \exp\left(-R_{\text{post}} t'\right)\right]. \tag{35}$$

Note that Eqs. 34 and 35 are used to fit the pre- and post-uncaging data for different target concentrations which leads to our finding a phenomenological suppression factor. Also note that, as written, these equations make no *a priori* assumptions about the existence of such a suppression factor which is independent of target concentration.

More generally, if the applied light dose only partially uncages the RNP population, R_{post} in Eq. 35 is replaced by the dose-dependent rate $R(H)$ from Eq. 29. To compare this model to the light-dose experiment in Fig. 1C, we first estimated a reaction rate for each illumination condition by fitting the reporter-cleavage time course to Eq. 33. The suppression factor Σ was fixed to the independently measured ratio between the fully uncaged and caged guide reaction rates, as found in our target concentration sweep experiments (main text, Fig. 2). The only remaining free parameter in Eq. 29 was therefore the effective photosensitivity $\alpha(\lambda)$, which determines how rapidly the caged RNP population is converted as cumulative light dose increases.

For each exposure time T, the applied dose was calculated as $H = I_{\text{LED}} T$, with $I_{\text{LED}} =$

1.6×10^{-3} J s^{-1} (measured with a ThorLabs PM100D2 hand-held power meter, see Fig. S4). Fitting Eq. 29 to the measured reaction rates gave $\alpha = 16.3 \pm 1.4$ J^{-1}. This corresponds to an uncaged fraction $f^u(H) = 1 - \exp[-\alpha H]$, giving estimated uncaged fractions of 0.23, 0.54, 0.79, and 0.96 after 10, 30, 60, and 120 s of illumination, respectively. The resulting dose-response closely matched the measured increase in reaction rate with light dose (Fig. 1C, main text), supporting the interpretation that the observed dose dependence is explained by first-order depletion of the caged RNP population.

2 Supplementary Figures

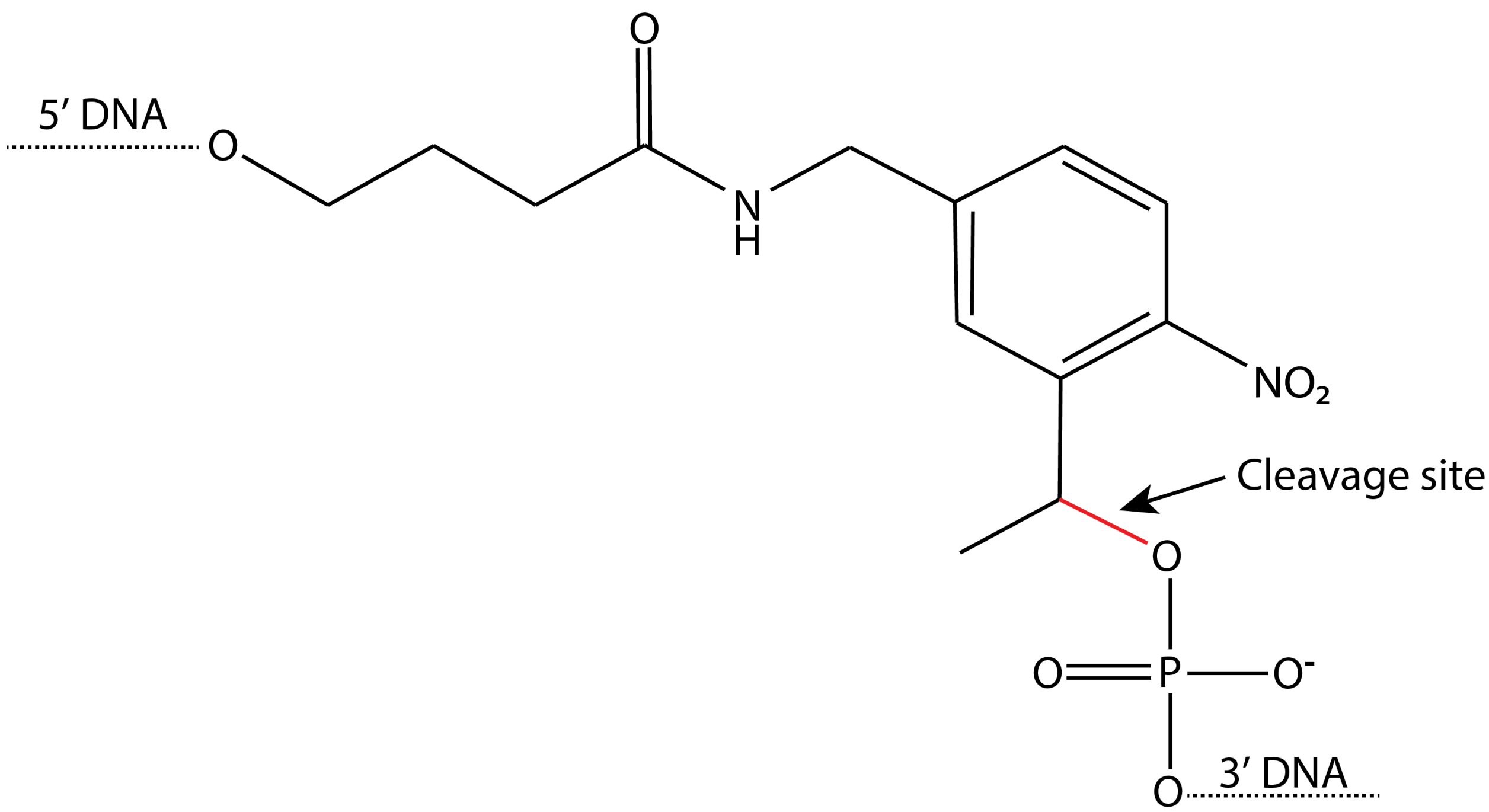

Supplementary Figure S1 Photocleavable linker chemical structure. (A) Photocleavable linker located in the DNA region of the interfering guide RNA (igRNA) to form a photocleavable interfering guide RNA (pc-igRNA).

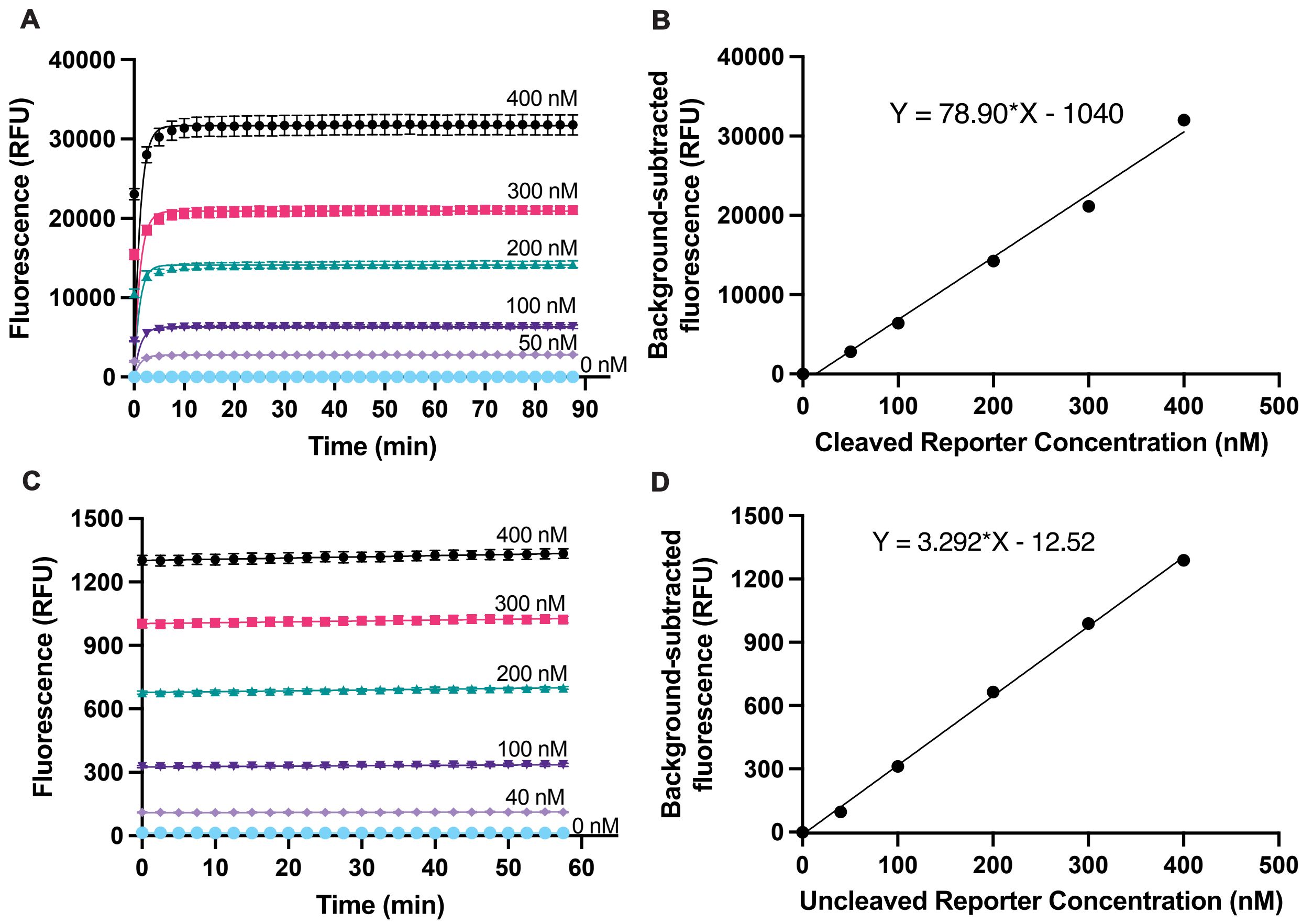

Supplementary Figure S2 Fluorescence versus cleaved and uncleaved reporter concentration calibration. **(A)** Raw measurements of fluorescence over time for LbuCas13a with gRNA 1 and 1×10^7 cp/μL of RNA target concentration. **(B)** Background-subtracted fluorescence versus cleaved reporter concentration with a linear regression fit. **(C)** Raw measurements of fluorescence over time for uncleaved reporter concentrations. **(D)** Background-subtracted fluorescence versus uncleaved reporter concentration with a linear regression fit. In all panels, the data represent the mean ± SEM across three replicates.

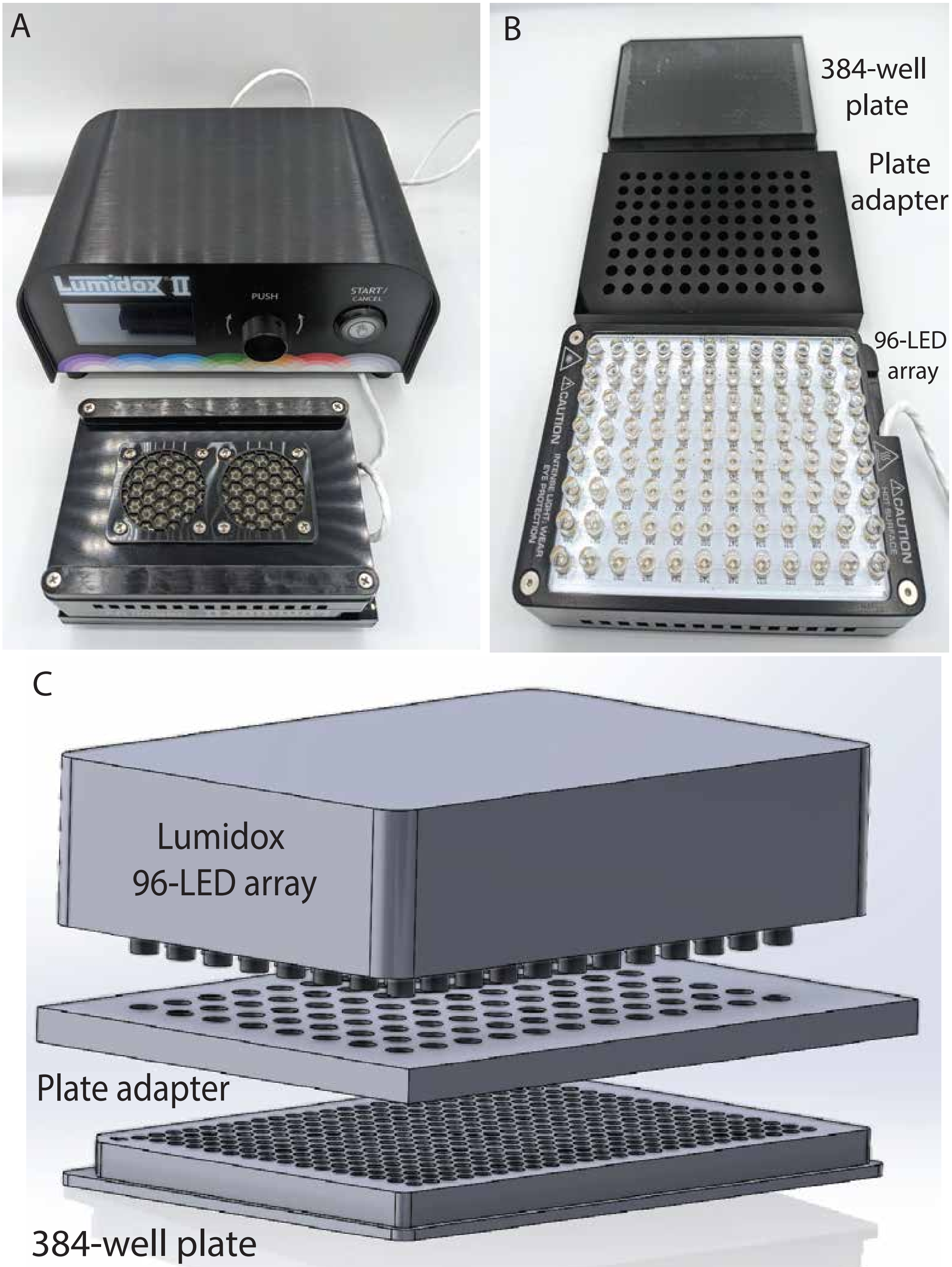

Supplementary Figure S3 LUCas assay illumination hardware. **(A)** Lumidox II Controller connected to the 96-LED array (365 nm). **(B)** 96-LED array, acrylic custom plate adapter and a 384 well plate. **(C)** The 96-LED array is placed on top of an acrylic custom plate adapter which aligns the individual LEDs directly above a single well from a 384-well plate. All non-exposed wells are used as caged wells.

A

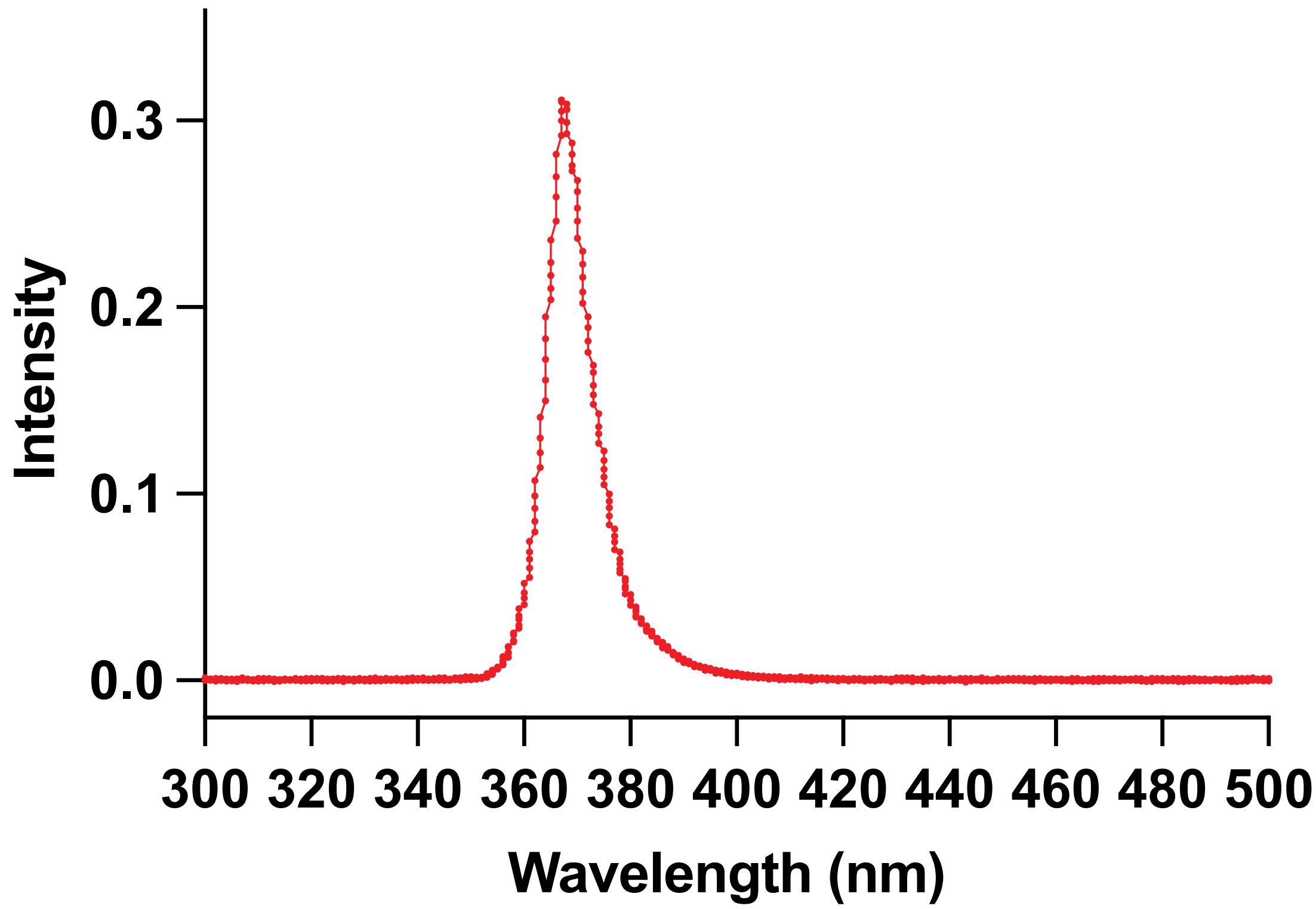

B

Power setting on Lumidox (mW)	Irradiance per well (mW/cm^2)	Measured Power at exposed well (mW)	Measured Power at non-exposed well (mW)
20	2.26	0.226	1.15e-3
40	5.70	0.570	2.23e-3
60	9.18	0.918	3.34e-3
80	12.70	1.270	4.46e-3
110	16.12	1.612	5.56e-3

Supplementary Figure S4 Characterization of the Lumidox LED array. (A) Spectrum of a single LED from the Lumidox 96-LED Array. **(B)** Summary of measured power for both exposed and non-exposed single wells in a 384 well plate.

A

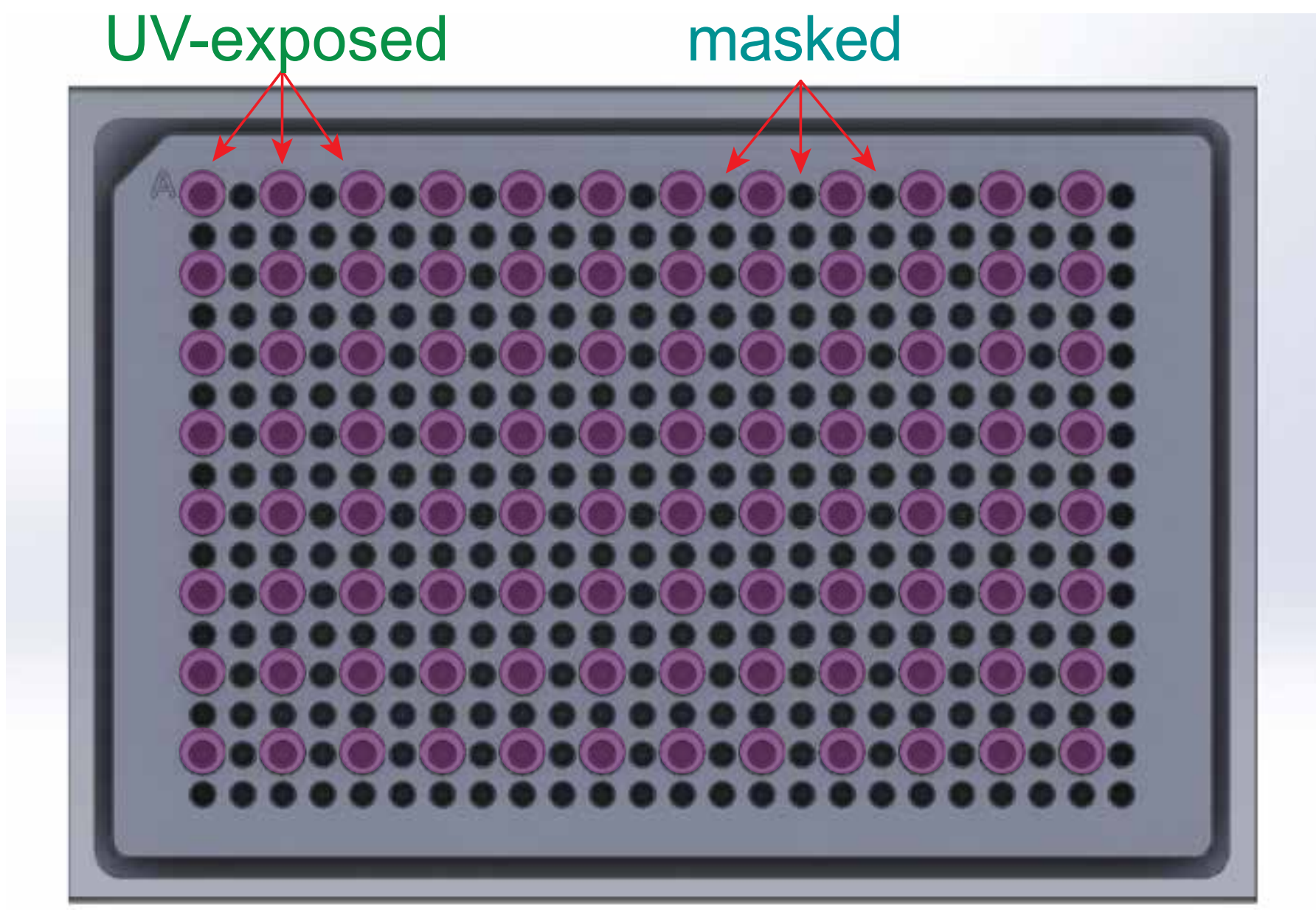

B

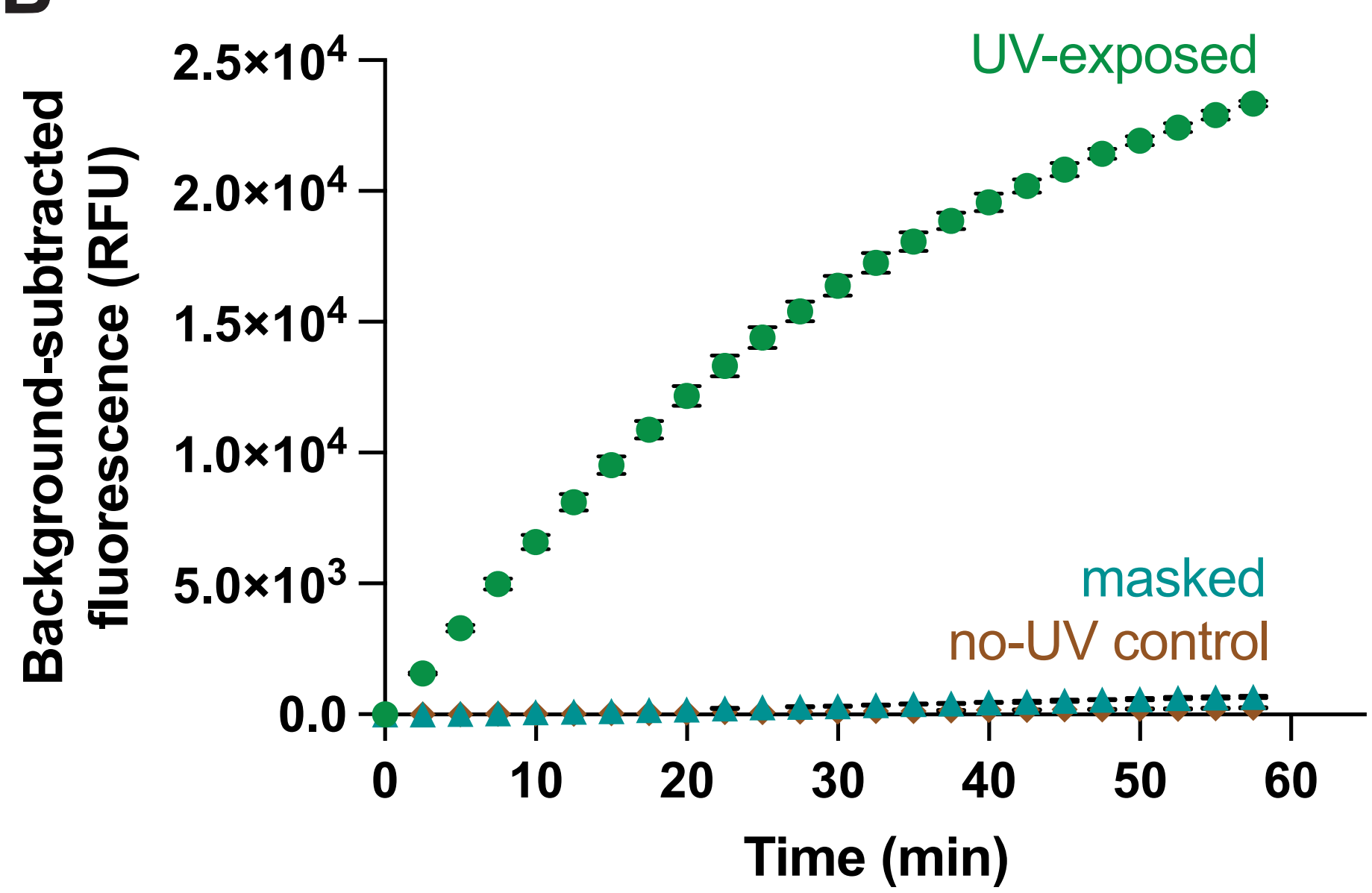

Supplementary Figure S5 Absence of *trans*-cleavage activity in masked wells. (A) Top view of the 384 well plate with the acrylic custom plate adapter. **(B)** CRISPR reactions with a target concentration (1×10^6 cp/μL) were mixed simultaneously. Reactions in masked wells were compared with reactions excluded from the LUCas illumination setup, which served as no-UV control wells. Data points represent the mean ± SEM (n=3).

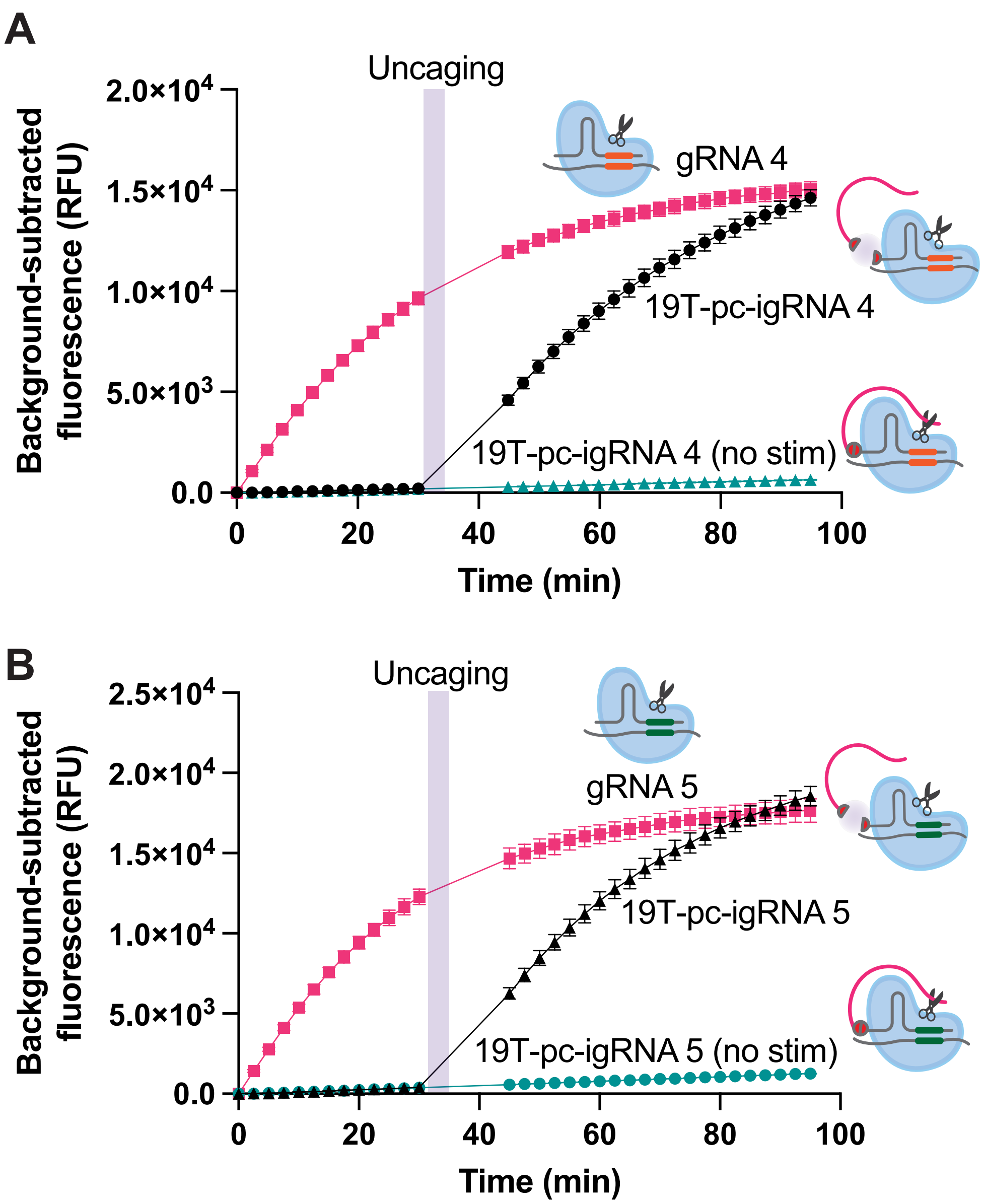

Supplementary Figure S6 Repeatability and reproducibility of LUCas guides. Fluorescence measurements over time were performed for two guides with different spacer regions: **(A)** 19T-pc-igRNA 4 **(B)** 19T-pc-igRNA 5. Data points represent the mean ± standard error of the mean (SEM) (n=3). The same RNA target concentration (1×10^6 cp/μL) was added for each condition.

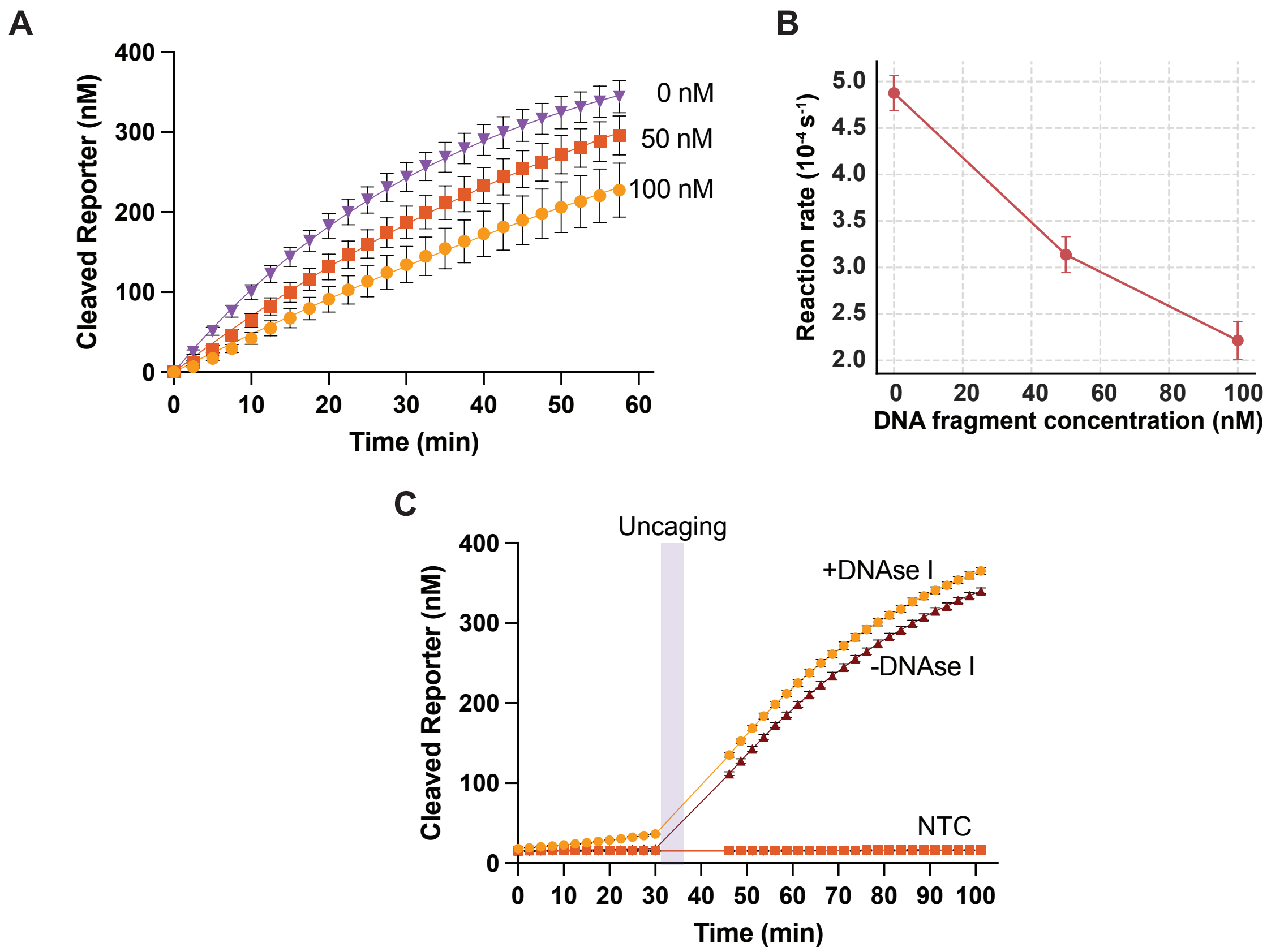

Supplementary Figure S7 Non-specific effects of short DNA fragments. **(A)** Performance of LbuCas13a-gRNA 1 RNP with 1×10^6 cp/μL of synthetic RNA target and different concentrations of free 20T fragments. **(B)** Reaction rates, extracted through a Michaelis-Menten kinetics fit, as a function of increasing concentration of the 20T fragments. **(C)** Comparison of Cas13a *trans*-cleavage activity with pc-igRNA 1 in the presence and absence of DNase I solution. In all panels, the data represents the mean ± SEM across three replicates.

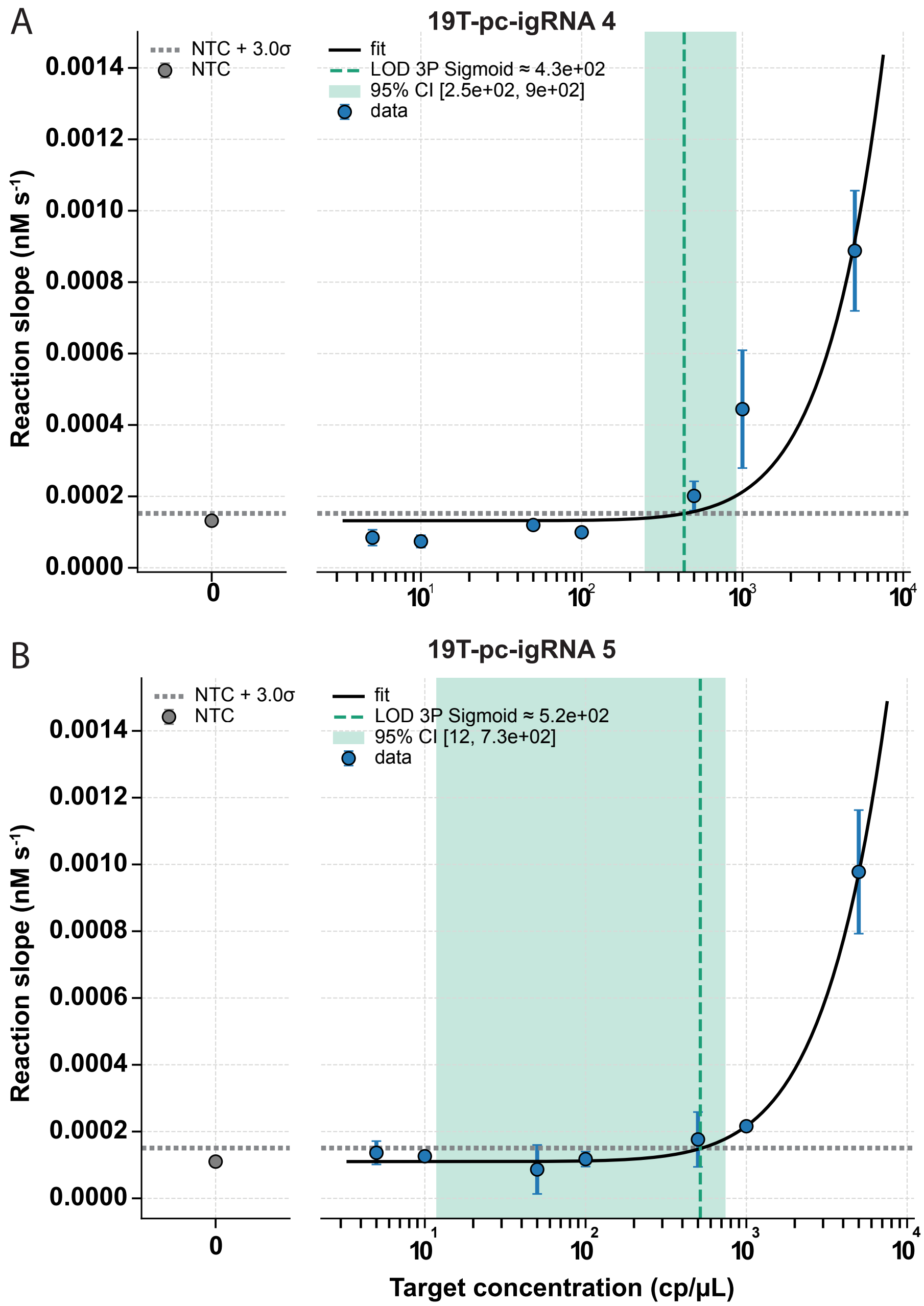

Supplementary Figure S8 Limit of detection analysis for other pc-igRNAs. (A) 19T-pc-igRNA 4 and **(B)** 19T-pc-igRNA 5 showing the measured background (NTC) (gray point); measured LOD in target concentration (vertical green dashed line) and 95% CI (green shaded region).

Supplementary Tables

Supplementary Table S1 Oligonucleotides used in this study.

Name	Sequence
gRNA 1	GACCACCCCAAAAAUGAAGGGGACUAAAAC**UUUGCGGCCAAUGUUUGUAA**
gRNA 2	GACCACCCCAAAAAUGAAGGGGACUAAAAC**GGUCCACCAAACGUAAUGCG**
gRNA 3	GACCACCCCAAAAAUGAAGGGGACUAAAAC**CAGUUGUGAUGAUUCCUAAG**
gRNA 4	GACCACCCCAAAAAUGAAGGGGACUAAAAC**AAGGUGUGACUUCCAUGCCA**
gRNA 5	GACCACCCCAAAAAUGAAGGGGACUAAAAC**AUUGGUGUUAAUUGGAACGC**
gRNA FluA	GACCACCCCAAAAAUGAAGGGGACUAAAAC**CAGCUGACAUGAGUAUUGGA**
19T-pc-igRNA 1	TTTTTTTTTTTTTTTTTTT/iSpPC/CGACCACCCCAAAAAUGAAGGGGACUAAAAC**UUUGCGGCCAAUGUUUGUAA**
9T-pc-igRNA 1	TTTTTTTTT/iSpPC/CGACCACCCCAAAAAUGAAGGGGACUAAAAC**UUUGCGGCCAAUGUUUGUAA**
9T-pc-igRNA 2	TTTTTTTTT/iSpPC/CGACCACCCCAAAAAUGAAGGGGACUAAAAC**GGUCCACCAAACGUAAUGCG**
9T-pc-igRNA 3	TTTTTTTTT/iSpPC/CGACCACCCCAAAAAUGAAGGGGACUAAAAC**CAGUUGUGAUGAUUCCUAAG**
19T-pc-igRNA 4	TTTTTTTTTTTTTTTTTTT/iSpPC/CGACCACCCCAAAAAUGAAGGGGACUAAAAC**AAGGUGUGACUUCCAUGCCA**

Name	Sequence
19T-pc-igRNA 5	TTTTTTTTTTTTTTTTTTT/iSpPC/CGACCACCCCAAAAAUGAAGGGGACUAAAAC**AUUGGUGUUAAUUGGAACGC**
gRNA 1 target	GGAACUGA**UUACAAACAUUGGCCGCAAA**UUGCACAA
gRNA 2 target	UGCACCC**CGCAUUACGUUUGGUGGACC**CUCAGAUU
gRNA 3 target	CUUGUUUU**CUUAGGAAUCAUCACAACUG**UAGCUGCA
gRNA 4 target	UCGCGCAU**UGGCAUGGAAGUCACACCUU**CGGGAACG
gRNA 5 target	AGGACAAG**GCGUUCCAAUUAACACCAAU**AGCAGUCC
PolyU Reporter	/56-FAM/UUUUU/3IABkFQ/
20T Fragment	TTTTTTTTTTTTTTTTTTTT
primer F	GAAATTAATACGACTCACTATAGGGAGACGTGGTCCAGAACAAACCCAAGGAAATT
primer R	TCTTTGTCATCCAATTTGATGGCACCTGTG

DNA is underlined. The 20-nucleotide sequence in bold is complementary to the gRNA spacer.

Supplementary Table S2 Overview of Light-Controlled CRISPR Diagnostic Strategies

**Publication**	**Cas Enzyme**	**Light Strategy**	**Key Advantages**
Jain et al. (2016) [1]	Cas9	"Protector" oligo, which contains at least 3 photocleavable groups that bind to the target region of a single chimeric guide RNA (sgRNA).	Quick irradiation (30 sec). Simultaneous or synchronized gene editing of three DNA targets.
Wang et al. (2020) [2]	LbuCas13a	Guide (crRNA) was masked using the DMNEC moiety	Masking protects crRNA from RNase degradation.
Chen et al. (2022) [3]	LbCas12a	Photocleavable complementary ssDNA (PC-DNA) to block the guide (crRNA).	Compatible with Recombinase Polymerase Amplification (RPA) for a one-pot reaction.
Hu et al. (2022) [4], Sun et al. (2024) [5]	LbCas12a and AsCas12a	Protective oligo that binds to the spacer region and partially binds 5 additional bases of the stem-loop region of the guide.	Combines RPA-Cas12a for a one-pot approach. Eliminates aerosol contamination.

Continued on next page

**Publication**	**Cas Enzyme**	**Light Strategy**	**Key Advantages**
Hu et al. (2023) [6], Pu et al. (2025) [7]	LbCas12a	Photolabile groups modifications (NPOM-dT) on the crRNA that prevent target binding.	The *trans*-cleavage activity can be tuned depending on the number of NPOMs.
Liu et al. (2024) [8], Du et al. (2026) [9]	LbCas12a	Photolabile groups (PLGs) bind to the stem-loop region of the guide RNA.	Acylation of crRNA with PLGs is a universal approach. Both *cis* and *trans* cleavage activities are blocked.
Li et al. (2024) [10], Cui et al. (2026) [11]	LbCas12a	A protector oligo that binds to the hairpin structure of a Cas12 crRNA. Uridine substitutions for NPOM-dT in the hairpin structure.	Detects human papillomavirus type 16 (HPV16) and *survivin* mRNA. This approach can be combined with lateral flow assays (LFA).
Wu et al. (2025) [12]	LbCas12a	Hybridization of a photocleavable ssDNA blocker using a 2-nitrobenzyl linker at three specific sites.	Enables on/off control of *trans*-cleavage activity. Develop a hierarchical temporal authorization system.

Continued on next page

**Publication**	**Cas Enzyme**	**Light Strategy**	**Key Advantages**
Hu et al. (2025) [13]	LbCas12a, BrCas12b and LbuCas13a	Photocleavable (PC) phosphorothioate (PS) DNA can occupy the target binding site and inhibit activity.	Combines a loop-mediated isothermal amplification (LAMP) with Cas12b for a one-pot reaction.
Zhuang et al. (2025) [14]	LbCas12a	Photocleavable (PC) DNA linker between the guide 3' terminus and the extension.	This approach only requires one PC spacer and 31–46 nt extension.
Yu et al. (2024) [15]	PspCas13	FKBP-FRB dimerization domains and the magnet system	In vivo validation in a mouse model. Reversible RNA base editing.
Tian et al. (2025) [16]	LbCas12a	Single-base photocaging at positions 3 and 4 of the crRNA repeat recognition sequence using NPOM-dT.	Validation of split crRNA design for cost-effective and flexible assay assembly.

Continued on next page

**Publication**	**Cas Enzyme**	**Light Strategy**	**Key Advantages**
Ng. and Krishnamurthy et al. (LU-Cas, this work)	LbuCas13a	Added a single PC linker between the guide 5' end and a polyT DNA sequence (pc-igRNA).	Requires only one PC spacer. The suppression and photo-activation are largely orthogonal to guide-target kinetics Requires <1 min of UV exposure.

References

[1] Piyush K. Jain, Vyas Ramanan, Arnout G. Schepers, Nisha S. Dalvie, Apekshya Panda, Heather E. Fleming, and Sangeeta N. Bhatia. Development of Light-Activated CRISPR Using Guide RNAs with Photocleavable Protectors. *Angewandte Chemie International Edition*, 55(40):12440–12444, September 2016.

[2] Shaoru Wang, Lai Wei, Jia-Qi Wang, Huimin Ji, Wei Xiong, Jian Liu, Ping Yin, Tian Tian, and Xiang Zhou. Light-Driven Activation of RNA-Guided Nucleic Acid Cleavage. *ACS Chemical Biology*, 15(6):1455–1463, June 2020.

[3] Yong Chen, Xiaoling Xu, Jiachun Wang, Yibin Zhang, Wentao Zeng, Yizhen Liu, and Xueji Zhang. Photoactivatable CRISPR/Cas12a Strategy for One-Pot DETECTR Molecular Diagnosis. *Analytical Chemistry*, 94(27):9724–9731, July 2022.

[4] Menglu Hu, Zhiqiang Qiu, Zirong Bi, Tian Tian, Yongzhong Jiang, and Xiaoming Zhou. Photocontrolled crRNA activation enables robust CRISPR-Cas12a diagnostics. *Proceedings of the National Academy of Sciences*, 119(26):e2202034119, June 2022.

[5] Qian Sun, Hongqing Lin, Yuan Li, Liping Yuan, Baisheng Li, Yunan Ma, Haiying Wang, Xiaoling Deng, Hongliang Chen, and Shixing Tang. A photocontrolled one-pot isothermal amplification and CRISPR-Cas12a assay for rapid detection of SARS-CoV-2 Omicron variants. *Microbiology Spectrum*, 12(3):e03645–23, March 2024.

[6] Menglu Hu, Ruhan Liu, Zhiqiang Qiu, Feng Cao, Tian Tian, Yunxin Lu, Yongzhong Jiang, and Xiaoming Zhou. Light-Start CRISPR-Cas12a Reaction with Caged crRNA Enables Rapid and Sensitive Nucleic Acid Detection. *Angewandte Chemie International Edition*, 62(23):e202300663, June 2023.

[7] Jing Pu, Shaolan Zhou, Wenxin Ma, Chang Liu, Le Guo, Bei Yan, and Zhenghao Huo. Light-controlled CRISPR–Cas12a one-pot platform for ultrasensitive cell-free DNA de-

tection in systemic lupus erythematosus diagnosis. *Analytical Methods*, 17(44):8916–8922, 2025.

[8] Pengfei Liu, Yating Lin, Xiaohua Zhuo, Jiayu Zeng, Bolin Chen, Zhen Zou, Guhuan Liu, Erhu Xiong, and Ronghua Yang. Universal crRNA Acylation Strategy for Robust Photo-Initiated One-Pot CRISPR–Cas12a Nucleic Acid Diagnostics. *Angewandte Chemie International Edition*, 63(23):e202401486, June 2024.

[9] Jinlian Du, Xingxiang Pu, Tongyan Yuan, Fang Peng, Jingjing Hu, Han Li, Bojie Chen, Jiaming Luo, Sheng Li, Yuling Teng, Xinyue Zhu, Weiming Chen, Qingqing Xie, Ling Jiang, Erhu Xiong, and Ronghua Yang. Plug-and-Play Photo-Initiated CRISPR–Cas12a One-Pot Nucleic Acid Detection via Universal Repeat RNA Acylation Strategy. *Analytical Chemistry*, 98(3):2136–2145, January 2026.

[10] Qing-Nan Li, Dong-Xia Wang, Dan-Ye Chen, Jia-Ao Lyu, Ya-Xin Wang, Shun-Li Wu, Hong-Xin Jiang, and De-Ming Kong. Photoactivatable CRISPR/Cas12a Sensors for Biomarkers Imaging and Point-of-Care Diagnostics. *Analytical Chemistry*, 96(6):2692–2701, February 2024.

[11] Jieyu Cui, Lulu Zhang, Jing Zhou, Ting Shi, Shangyi Wu, Tenghui Dai, Lijun Hao, Junjie Pan, Xiaodan Lai, Wentao Lu, Xingxu Huang, Zhanjun Li, Liangxue Lai, and Xinjie Wang. A universal light-controlled highly sensitive one-pot CRISPR/Cas12a diagnostic based on structure-engineered crRNA. *Trends in Biotechnology*, page S0167779926001253, April 2026.

[12] Jia-ni Wu, Changjiang Li, Zhujun Liu, Xiaolong Li, Jiarun Wang, Xiaoding Lou, Fan Xia, Jun Dai, and Tongbo Wu. Photocontrolled dissociation and toehold-mediated strand displacement-based synergistic regulation of CRISPR-Cas12a. *Nucleic Acids Research*, 53(20):gkaf1178, October 2025.

[13] Menglu Hu, Bingni Zhang, Yuanyue Shan, Feng Cao, Yihui Wang, Weiwei Qi, Xue Wang, Yuting Shen, Xinyi Guo, Mengmeng Zhang, Tian Tian, Wei Xie, Mingfeng Zhang, Fang Liang, Duanqing Pei, and Xiaoming Zhou. Scalable modulation of CRISPR–Cas enzyme activity using photocleavable phosphorothioate DNA. *Nature Communications*, 16(1):5939, July 2025.

[14] Songkuan Zhuang, Yueyu Dong, Xiaojun Huang, Tonggong Liu, Dayong Gu, and Yizhen Liu. A universal Cas12a activity regulation strategy via photocleavable crRNA 3'-terminal additives enables one-pot CRISPR detection of arboviruses. *Chemical Engineering Journal*, 521:166699, October 2025.

[15] Jeonghye Yu, Jongpil Shin, Jihwan Yu, Jihye Kim, Daseuli Yu, and Won Do Heo. Programmable RNA base editing with photoactivatable CRISPR-Cas13. *Nature Communications*, 15(1):673, January 2024.

[16] Tian Tian, Hongrui Xiao, Xinyi Guo, Yuxin Chen, Zhiqiang Qiu, Ting Zhang, Meiyu Chen, Weiwei Qi, Peige Cai, Meng Cheng, and Xiaoming Zhou. Identification of a key nucleotide influencing Cas12a crRNA activity for universal photo-controlled CRISPR diagnostics. *Nature Communications*, 16(1):6694, July 2025.
